# Heparin impairs skeletal muscle glucose uptake by inhibiting insulin binding to insulin receptor

**DOI:** 10.1002/edm2.253

**Published:** 2021-05-05

**Authors:** Canjun Zhu, Zhiyue Xu, Yexian Yuan, Tao Wang, Chang Xu, Cong Yin, Peipei Xie, Pingwen Xu, Hui Ye, Nirali Patel, Sarah Schaul, Lina Wang, Xiaotong Zhu, Songbo Wang, Ping Gao, Qianyun Xi, Yongliang Zhang, Gang Shu, Qingyan Jiang

**Affiliations:** ^1^ Guangdong Laboratory of Lingnan Modern Agriculture Guangdong Province Key Laboratory of Animal Nutritional Regulation and National Engineering Research Center for Breeding Swine Industry College of Animal Science South China Agricultural University Guangzhou China; ^2^ Huadong Sanatorium Wuxi China; ^3^ Division of Endocrinology, Diabetes and Metabolism Department of Medicine The University of Illinois at Chicago Chicago IL USA

**Keywords:** GLUT4 activity, heparin, hyperglycaemia, insulin resistance, muscle glucose uptake

## Abstract

**Aim:**

Heparin, a widely used antithrombotic drug has many other anticoagulant‐independent physiological functions. Here, we elucidate a novel role of heparin in glucose homeostasis, suggesting an approach for developing heparin‐targeted therapies for diabetes.

**Methods:**

For serum heparin levels and correlation analysis, 122 volunteer’s plasma, DIO (4 weeks HFD) and db/db mice serums were collected and used for spectrophotometric determination. OGTT, ITT, 2‐NBDG uptake and muscle GLUT4 immunofluorescence were detected in chronic intraperitoneal injection of heparin or heparinase (16 days) and muscle‐specific loss‐of‐function mice. In 293T cells, the binding of insulin to its receptor was detected by fluorescence resonance energy transfer (FRET), Myc‐GLUT4‐mCherry plasmid was used in GLUT4 translocation. In vitro, C2C12 cells as mouse myoblast cells were further verified the effects of heparin on glucose homeostasis through 2‐NBDG uptake, Western blot and co‐immunoprecipitation.

**Results:**

Serum concentrations of heparin are positively associated with blood glucose levels in humans and are significantly increased in diet‐induced and *db/db* obesity mouse models. Consistently, a chronic intraperitoneal injection of heparin results in hyperglycaemia, glucose intolerance and insulin resistance. These effects are independent of heparin’s anticoagulant function and associated with decreases in glucose uptake and translocation of glucose transporter type 4 (GLUT4) in skeletal muscle. By using a muscle‐specific loss‐of‐function mouse model, we further demonstrated that muscle GLUT4 is required for the detrimental effects of heparin on glucose homeostasis.

**Conclusions:**

Heparin reduced insulin binding to its receptor by interacting with insulin and inhibited insulin‐mediated activation of the PI3K/Akt signalling pathway in skeletal muscle, which leads to impaired glucose uptake and hyperglycaemia.

## Graphical abstract

Circulating heparin is positively correlated with blood glucose level in humans and increases in diet‐induced obese and type 2 diabetic db/db mice. Chronic intraperitoneal injection of heparin results in hyperglycemia, glucose intolerance, and insulin resistance. Heparin interacts with insulin to inhibit insulin and insulin receptor binding in skeletal muscle, and by doing so, impairs skeletal muscle GLUT4 translocation and glucose uptake.

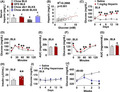

## 1 INTRODUCTION

Type 2 Diabetes mellitus (T2DM) is a ‘silent disease’ characterized by hyperglycaemia and reduced insulin function. It has become one of the most life‐threatening diseases of human health.^1^ While developing more effective treatments for T2DM is urgently needed, our understanding of the pathophysiology of T2DM is limited. Glycosaminoglycans (GAGs), the most abundant linear heteropolysaccharides in the body, play an essential role in glucose homeostasis regulation.^2^ Early studies have found a significant increase of GAGs levels in serum^3^ and urine^4^ in patients with diabetes. A recent study consistently indicated that the content of GAGs in urine is a potential diagnostic indicator for diabetes.^5^

Heparin, a naturally occurring GAG mainly stored within mast cells^6^ has been widely used in the clinical setting as an anticoagulant and antithrombotic drug.^7^ Notably, heparin has long been of interest in relation to the treatment of T2DM, particularly with the mitigation of renal and cardiovascular complications. In both human and animal studies, heparin has shown to prevent the occurrence of diabetic nephropathy,^8^ accelerate wound healing,^9^ and reduce the risk of thrombosis in diabetes mellitus.^10^ However, the limitations of heparin usage in diabetes have also been reported. For example, despite its beneficial anticoagulant functions during islet transplantation, heparin enhances islet amyloid deposition, thus affecting transplant success rate.^11^ Heparin treatment also results in aldosterone suppression and hyperkalaemia, particularly in patients with diabetes mellitus.^12^

Besides well‐established anticoagulant functions, various other physiological effects of endogenous heparin have been explored, for example anti‐inflammation,^13^ increasing lipid metabolism,^14^ stimulating appetite^15^ and promoting activity of insulin‐like growth factor‐1.^16^ Interestingly, it has been shown that blood levels of heparin in diabetic patients are higher than normal.^3^ Consistent with this association, a direct link between heparin and insulin signalling has been reported back in the 1980s. This pioneering study demonstrated reduced insulin function in the presence of heparin,^17^ suggesting a key role of heparin in glucose homeostasis. However, it was noted that this change occurred in specific tissues. The heparin‐induced inhibition of insulin binding was only observed in cultured human lymphocytes, but not in adipocytes, erythrocytes or hepatoma cells.^17^ Despite no changes in insulin binding, in isolated adipocytes, heparin reduced basal and insulin‐stimulated glucose oxidation. Additionally, in intact hepatoma cells, heparin inhibited both insulin binding and insulin‐stimulated autophosphorylation in receptors solubilized from these cells.^17^ These observations suggest a tissue‐specific role of heparin in insulin signalling and glucose homeostasis. Until now, there has been no direct evidence for the systemic effects of heparin on glucose homeostasis. The central questions of our study are whether heparin regulates whole‐body glucose homeostasis and whether it occurs through a tissue‐specific reduction of affinity for insulin.

In the present study, we first evaluated the physiological significance of circulating heparin in diabetic patients, *db/db* mice and diet‐induced obesity mice. We then investigated the systemic effects of heparin treatment on blood glucose, whole‐body glucose tolerance, whole‐body insulin sensitivity and glucose uptake in different tissues. Finally, we used a heparin agarose competitive binding assay, fluorescence resonance energy transfer (FRET) microscopy and a muscle‐specific conditional mouse model generated by the clustered regularly interspaced short palindromic repeats (CRISPR) technique to determine whether heparin impairs whole‐body glucose homeostasis by inhibiting insulin binding and subsequent actions in skeletal muscle.

## 2 RESEARCH DESIGN AND METHODS

### 2.1 Animals

All mice were housed at 20℃‐26℃, on a 12 h light‐dark cycle (6 a.m. and 6 p.m.). All mice within each experiment were of the same strain and sex, and had similar body weight (at the age of 10‐18 weeks). Unless otherwise stated, the mice were maintained ad libitum on standard mouse chow and water.

### 2.2 Heparin’s effects on glucose homeostasis

The systemic effects of heparin on glucose homeostasis were evaluated in C57BL/6J (BL6), *db/db*, muscle‐specific glucose transporter type 4 (GLUT4) KO and streptozotocin (STZ)‐induced type 1 diabetes mellitus (T1DM) mouse models. Blood glucose was measured once per four days in mice intraperitoneally (i.p.) injected with saline or 1 mg/kg heparin every other day for 16 days. Glucose tolerance, insulin sensitivity, and glucose uptake in epididymal white adipose tissue (EWAT), gastrocnemius (GST) and liver were measured after 16 days of heparin treatment.

### 2.3 Cellular signal mechanisms of heparin‐induced inhibition on skeletal muscle glucose uptake

Heparin’s effects on insulin‐insulin receptor interaction were tested by a heparin agarose competitive binding assay and fluorescence resonance energy transfer (FRET) microscopy. In both *in vitro* C2C12 cells and *in vivo* GST muscle of BL6 mice, glucose uptake was evaluated after co‐treatment of heparin and insulin, and heparin’s effects on insulin‐dependent downstream cascades were tested. To determine if heparin‐induced inhibition on muscle glucose uptake is mediated through classic insulin cascades, glucose uptake was monitored after heparin treatment in mutant mice with muscle‐specific deletion of GLUT4 or overexpression of protein kinase B (Akt).

### 2.4 Statistics

Statistical analyses were performed using GraphPad Prism. Methods of statistical analyses were chosen on the basis of the design of each experiment and are indicated in the figure legends. Data are presented as mean ± SEM. A *p*‐value less than .05 was considered to indicate statistical significance.

## 3 RESULTS

### 3.1 Heparin treatment impairs glucose homeostasis

**FIGURE 1:**
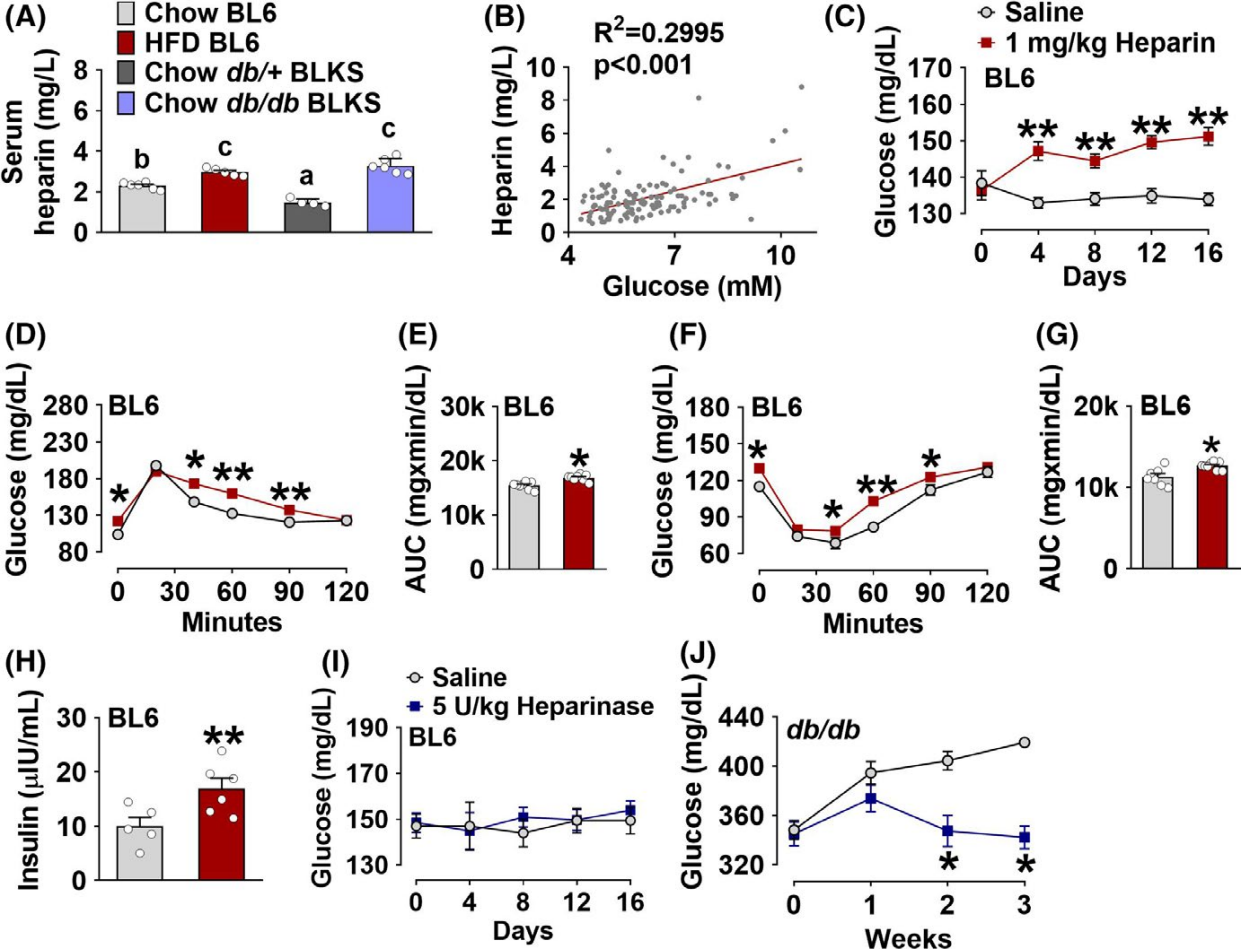
A, Serum heparin concentrations in male C57BL/6J (BL6) mice after 4 weeks chow diet or high‐fat diet (HFD) feeding (*n* = 5 or 6) and male *db*/+ or *db/db* littermates with C57BLKS/J (BLKS) background (*n* = 4 or 6). B, Linear correlation between blood glucose and heparin levels in humans (*n* = 122). C, Blood glucose of male BL6 mice intraperitoneally (i.p.) injected with saline or 1 mg/kg heparin every other day for 16 days (*n* = 7). D‐E, Oral glucose tolerance test (OGTT, D) and quantification of area under the curve (AUC, E) in male BL6 mice after 16 days of i.p. injection of saline or 1 mg/kg heparin (*n* = 7). F‐G, Insulin tolerance test (ITT, F) and quantification of AUC (G) in male BL6 mice after 16 days of i.p. injection of saline or 1 mg/kg heparin (*n* = 7). H, Serum insulin levels in male BL6 mice after 16 days of i.p. injection of saline or 1 mg/kg heparin (*n* = 5 or 6). I, Blood glucose of chow‐fed male BL6 mice i.p. injected with saline or 5 U/kg heparinase every other day for 16 days (*n* = 6). J, Blood glucose of male *db/db* BLKS mice i.p. injected with saline or 5 U/kg heparinase every other day for 3 weeks (*n* = 5). C, D, F, I and J, Results are presented as mean ± SEM. *, *p* ≤ .05, **, *p* ≤ .01 in two way ANOVA analyses followed by post hoc Bonferroni tests. (A, E, G and H) Results are presented as mean ± SEM. *, *p* ≤ .05 or **, *p* ≤ .01 in non‐paired Student's *t* test

Consistent with the high blood heparin levels observed in diabetic patients,^3^, ^18^ we showed that serum heparin levels were significantly elevated in both genetic and diet‐induced obesity mouse models. Specifically, we found that after 4 weeks of high‐fat diet feeding, serum heparin concentration in male C57BL/6J (BL6) mice was increased by 30% compared with that in chow‐fed control male BL6 mice (2.30 ± 0.17 vs 2.97 ± 0.19; Figure 1A). Consistently, male *db/db* (leptin receptor deficiency) C57BLKS/J (BLKS) mice, the most widely used mouse model of T2DM, had a similar but more robust increase of serum heparin levels compared to littermates with one allele of mutant in leptin receptor (*db*/+; 1.48 ± 0.18 vs 3.28 ± 0.37; Figure 1A). Notably, male *db*/+ BLKS mice showed significantly lower serum heparin levels compared with both chow‐ and HFD‐fed male BL6 mice. This inconsistency may be attributed to the difference in background strain. It has been shown that despite 70% genetic homology, BL6 and BLKS inbred mouse strains differ dramatically in response to diet‐ and genetic‐induced obesity.^19^, ^20^ Interestingly, plasma heparin concentrations in humans also exhibited a statistically significant positive correlation with blood glucose levels (Figure 1B), suggesting an essential role of heparin in blood glucose control.

The elevated blood heparin in both obese animals and diabetic patients leads a notion that aberrant levels of heparin may contribute to glucose homeostasis disorders. To test this possibility, we intraperitoneally (i.p.) injected heparin (1 mg/kg) in chow‐fed male BL6 mice every other day for 16 days. We found that chronic heparin treatment induced a significant increase in blood glucose compared to control littermates injected with saline (Figure 1C). The stimulatory effects of heparin were observed as early as 4 days after injection and lasted for the duration of the experiment (Figure 1D). Consistently, glucose intolerance (Figure 1D,E) and insulin resistance (Figure 1F,G) were observed after 16 days of heparin injection, indicating impaired glucose homeostasis. Additionally, 16 days heparin treatment significantly increased serum levels of insulin (Figure 1H) but not glucagon or corticosterone (Figure S1A,B), suggesting an impaired insulin action.

The essential role of heparin in glucose homeostasis maintenance is further supported by the evidence from mice chronically treated with heparinase, a key enzyme specifically catalysing the degradation of heparin. We found that while heparinase failed to decrease blood glucose of chow‐fed male BL6 mice (Figure 1I), it dramatically reduced the blood glucose of diabetic *db/db* mice after 3 weeks of treatment (Figure 1J). These data suggest a key role of heparin in *db/db*‐induced diabetes. These observations are consistent with indirect evidence regarding another member of the GAG family, heparan sulphate, which shares a very similar structure with heparin. Heparanase, the catalytic enzyme of heparin sulphate, has been shown to effectively alleviate genetic and chemical‐induced T1DM in mice.^21^, ^22^ Additionally, we tested the blood glucose levels after chronic treatment of two other heparin derivatives, desulphated heparin (DSH with lower anticoagulant activity) and low‐molecular‐weight heparin (LMWH with higher anticoagulant activity). We found that DSH induced a similar increase in blood glucose levels as we observed in heparin‐treated mice (Figure S1C), whereas LMWH failed to affect blood glucose levels (Figure S1D). Hence, heparin‐induced hyperglycaemia is likely independent of its anticoagulant activity.

### 3.2 Heparin reduces glucose uptake and GLUT4 translocation in skeletal muscle

**FIGURE 2:**
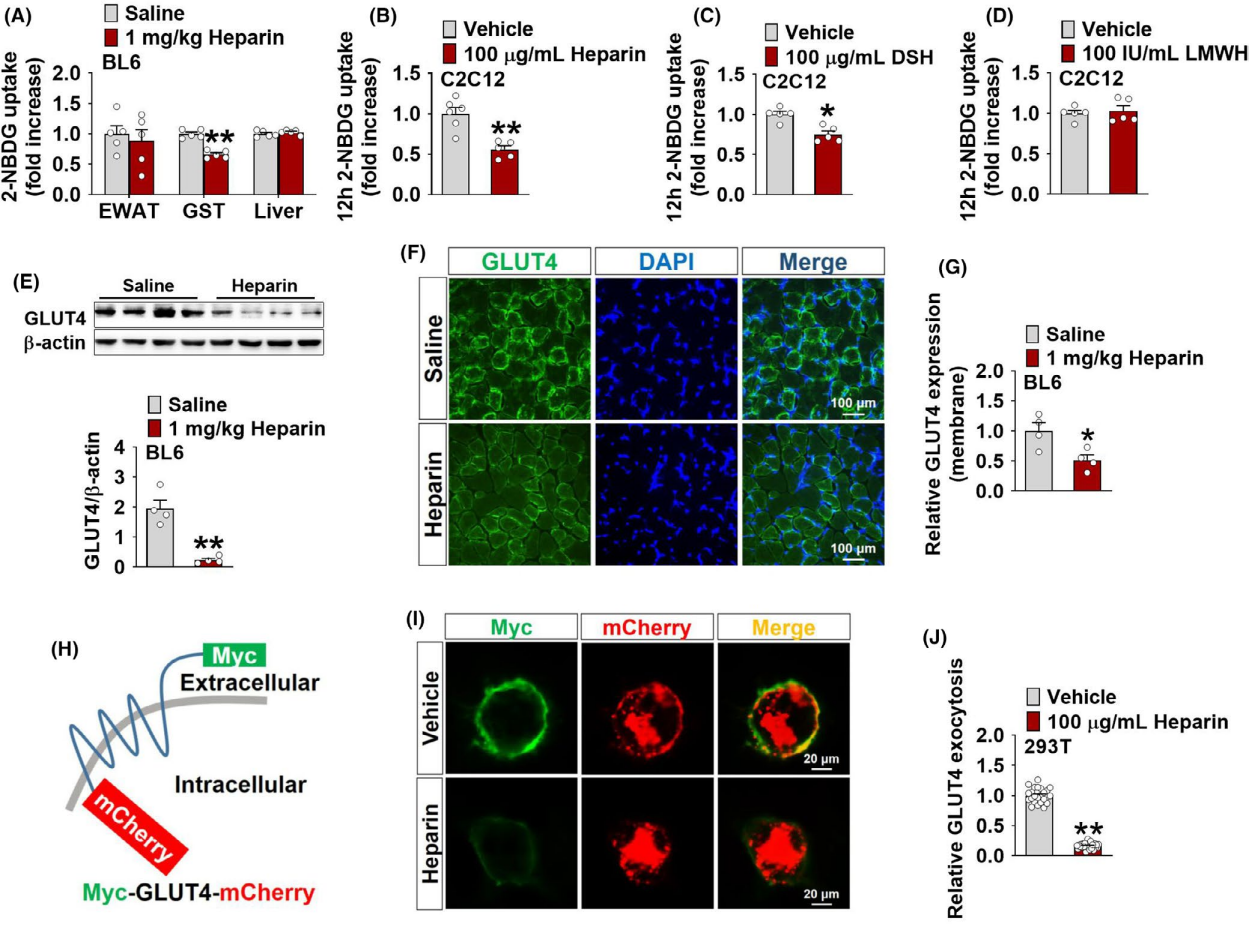
A, Uptake of 2‐Deoxy‐2‐[(7‐nitro‐2,1,3‐benzoxadiazol‐4‐yl) amino]‐D‐ glucose (2‐NBDG) in the epididymal white adipose tissue (EWAT), gastrocnemius muscle (GST) and liver of male BL6 mice after i.p. injection of saline or 1 mg/kg heparin every other day for 16 days (*n* = 5). B‐D, Uptake of 2‐NBDG in C2C12 cells cultured with vehicle or 100 μg/ml heparin (B), vehicle or 100 μg/ml DSH (C) and vehicle or 100 IU/ml LMWH (D) (control data in this panel same as C) for 12 h (*n* = 5 or 6), vehicle (cell culture medium). E, Immunoblots and quantification of glucose transporter type 4 (GLUT4) protein in the GST of male BL6 mice after i.p. injection of saline or 1 mg/kg heparin every other day for 16 days (*n* = 4). F‐G, Representative images (F) and quantification (G) of GLUT4 membrane protein in the GST of male BL6 mice after i.p. injection of saline or 1 mg/kg heparin every other day for 16 days. GLUT4 was stained with green fluorescence. DAPI (blue) was used to mark the cell nuclei (*n* = 4). H, Schematic experimental strategy using Myc‐GLUT4‐mCherry fusion protein to detect GLUT4 translocation and plasma membrane surface exposure. I‐J, Representative images (I) and quantification (J) of GLUT4 surface exposure in 293T cells cultured with vehicle or 100 μg/ml heparin for 12 h. The ratio of surface to total GLUT4 was quantified by dividing Myc green fluorescence to mCherry red fluorescence. Data were normalized and expressed as a percentage of vehicle‐treated cells (*n* = 24), vehicle (cell culture medium). Results are presented as mean ± SEM. *, *p* ≤ .05 or **, *p* ≤ .01 in non‐paired Student's *t* test

To explore the tissue‐specific mechanisms for heparin‐induced hyperglycaemia, we used a fluorescent derivative of glucose, 2‐Deoxy‐2‐[(7‐nitro‐2,1,3‐benzoxadiazol‐4‐yl)amino]‐D‐glucose (2‐NBDG), to measure glucose uptake^23^ in different tissues after chronic heparin treatment in chow‐fed male BL6 mice. These tissues include EWAT, GST and liver. All of these tissues have been known to play important roles in glucose uptake. Skeletal muscle and adipose tissue account for approximately 70% and 10% of insulin‐dependent glucose uptake, respectively, whereas liver acts as a site for insulin‐independent intracellular glucose uptake.^24^ Interestingly, we found that heparin decreased glucose uptake in GST but not in EWAT or liver (Figure 2A), suggesting a muscle‐specific impairment of glucose uptake induced by heparin. Consistently, heparin and DSH, but not LMWH, showed similar inhibitory effects on glucose uptake in *in vitro* C2C12 cells, an immortalized mouse myoblast cell line (Figure 2B‐D). Additionally, we also found that heparin failed to affect the mRNA expression of glycolysis‐related genes in the GST (Figure S2A) as well as glycolysis‐ or gluconeogenesis‐related genes in the liver (Figure S2B,C), indicating unchanged glycolysis and gluconeogenesis. These results suggest that heparin may induce hyperglycaemia by impairing skeletal muscle glucose uptake.

Insulin‐dependent glucose uptake in skeletal muscle is primarily mediated by altering the subcellular distribution of GLUT4 from intracellular stores to the plasma membrane, a process known as GLUT4 translocation.^25^ We asked whether GLUT4 translocation is altered by heparin signals. We found that chronic heparin treatment significantly decreased protein expression (Figure 2E) and plasma membrane distribution (Figure 2F,G) of GLUT4 in the GST of chow‐fed male BL6 mice, suggesting a heparin‐induced inhibition on GLUT4 translocation. This view is further supported by the evidence from an *in vitro* GLUT4 translocation cell model. Although HEK293 cells do not express insulin‐responsive GLUT4, insulin still could promote glucose uptake after artificial expression of GLUT4.^26^ So, we specifically chose 293T cells were transfected with a plasmid vector to express a Myc‐GLUT4‐mCherry fusion protein.^27^ In this model, Myc tag was inserted in the first exofacial loop of GLUT4 N terminus, and mCherry was fused at the C terminus (Figure 2H), which allows detection of GLUT4 on plasma membrane (by Myc in a non‐permeabilized condition) and total GLUT4 (by mCherry). We found that compared with vehicle‐treated cells, heparin‐treated cells showed a reduced plasma membrane distribution of GLUT4 (Figure 2I,J). These results suggest a possible role of GLUT4 in the inhibitory effects of heparin on muscle glucose uptake.

### 3.3 Skeletal muscle GLUT4 mediates heparin‐induced hyperglycaemia

**FIGURE 3:**
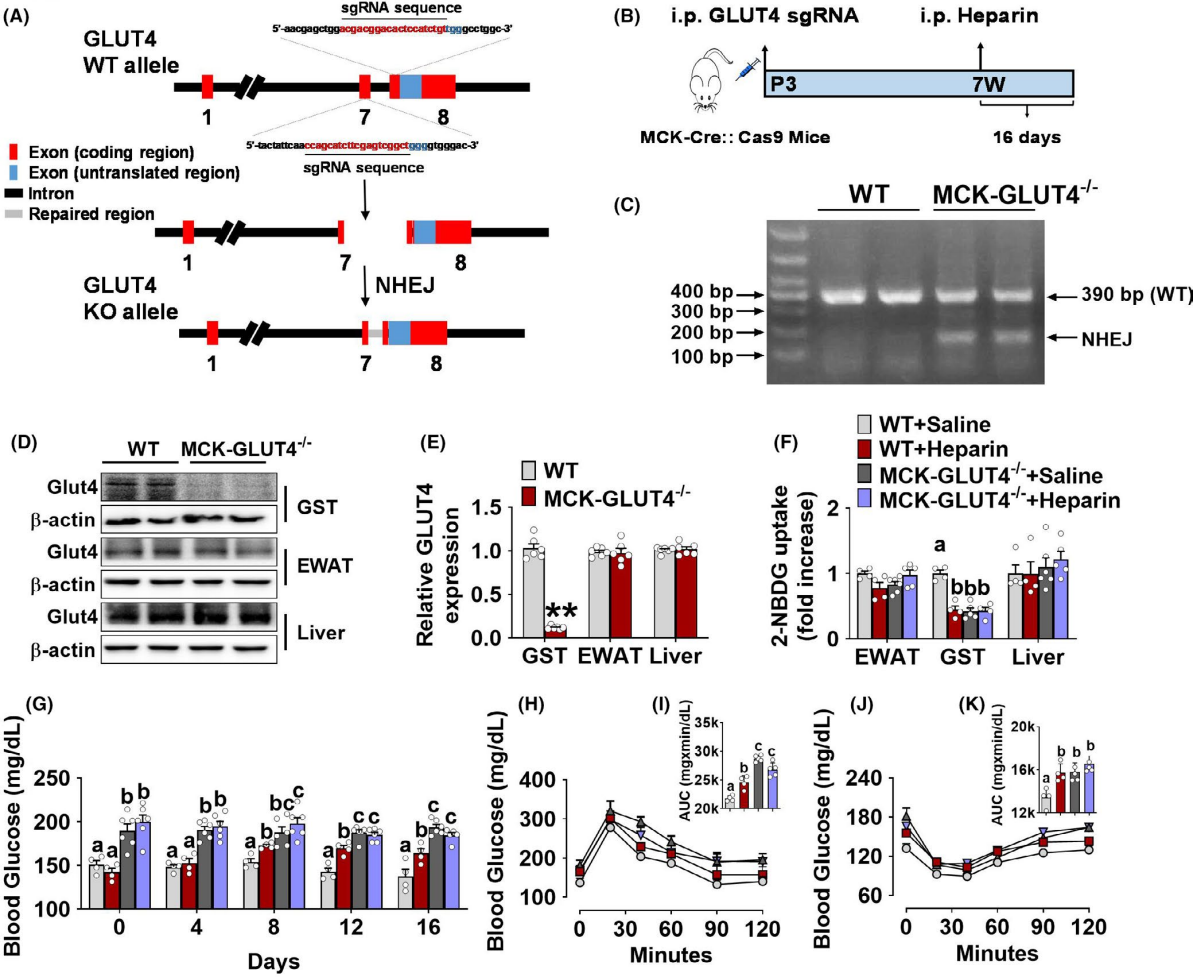
A, Schematic experimental strategy of Clustered Regularly Interspaced Short Palindromic Repeats (CRISPR) with sgRNAs targeting GLUT4 gene. The sgRNA sites were located in intron 7 and exon 8 of the GLUT4 gene. The DNA sequences contained sgRNA‐binding regions are labelled with red. NHEG, non‐homologous end joining. B, Schematic experimental strategy of generating muscle‐specific GLUT4 knockout mice. C, Genomic validation of GLUT4 knockout in the GST of male MCK‐GLUT4^−/−^ mice. D‐E, Immunoblots (D) and quantification (E) of GLUT4 protein in the GST, EWAT and liver of male MCK‐GLUT4^−/−^ mice (*n* = 6). F, Uptake of 2‐NBDG in the GST, EWAT and liver of male BL6 control or MCK‐GLUT4^−/−^ mice after i.p. injection of saline or 1 mg/kg heparin every other day for 16 days (*n* = 4 or 5). G, Blood glucose of male BL6 control or MCK‐GLUT4^−/−^ mice after i.p. injection of saline or 1 mg/kg heparin every other day for 16 days (*n* = 4 or 5). H‐I, OGTT (H) and quantification of AUC (I) in male BL6 or MCK‐GLUT4^−/−^ mice after i.p. injection of saline or 1 mg/kg heparin every other day for 16 days (*n* = 4 or 5). J‐K, ITT (J) and quantification of AUC (K) in male BL6 or MCK‐GLUT4^−/−^ mice after i.p. injection of saline or 1 mg/kg heparin every other day for 16 days (*n* = 4 or 5). E, Results are presented as mean ± SEM. **, *p* ≤ .01 in non‐paired Student's *t* test. (F, G, I and K) Results are presented as mean ± SEM, different letters between bars indicate *p* ≤ .05 by one‐way ANOVA followed by post hoc Tukey’s tests

To directly test if skeletal muscle GLUT4 is required for heparin‐induced hyperglycaemia, we generated a mouse model with GLUT4 selectively deleted in skeletal muscle by using the Clustered Regularly Interspaced Short Palindromic Repeats (CRISPR) method. Specifically, we crossed muscle creatine kinase‐Cre (MCK‐Cre) mice^28^ with Cre‐dependent Cas9 overexpression (LSL‐Cas9) mice^29^ to generate MCK‐Cre/LSL‐Cas9 (MCK‐Cas9) mice, a mouse model with Cas9 selectively over‐expressed in MCK positive muscle cells. As previously described,^30^ AAV virus vector carrying paired single guide RNAs (AAV1‐sgRNAs) targeting GLUT4 (sgRNA‐binding sites with 205bp interval were located in intron 7 and exon 8 of the GLUT4 gene) was then i.p. injected into MCK‐Cas9 mice at postnatal day 3 (P3) to generate a muscle‐specific GLUT4 deletion mouse model (MCK‐GLUT4^−/−^, Figure 3A,B). We found non‐homologous end joining (NHEJ) repair of GLUT4 in the GST of MCK‐GLUT4^−/−^ mice (Figure 3C), suggesting a Cas9‐induced GLUT4 DNA editing. Importantly, both GLUT4 mRNA and protein were dramatically decreased in the GST but not in the EWAT and liver of MCK‐GLUT4^−/−^ mice (Figure 3D,E), which validates our knockout model. In addition, we did not find any side effects during the experiment after virus injection, and only GFP expression was seen in muscle of MCK‐Cas9 mice (Figure S3A,B).

After validation, we investigated the effects of chronic heparin treatment on glucose homeostasis in MCK‐GLUT4^−/−^ mice. Consistent with previous reports,^31^, ^32^, ^33^ saline‐treated male MCK‐GLUT4^−/−^ mice showed impaired muscle glucose uptake, hyperglycaemia, glucose intolerance and insulin resistance (Figure 3F‐K) compared to saline‐treated male BL6 mice. This physiological evidence provides additional support for the successful deletion of muscle GLUT4 in MCK‐GLUT4^−/−^ mice. Notably, muscle‐specific GLUT4 knockout abolished the inhibitory effects of heparin on glucose uptake in the GST (Figure 3F), suggesting a key role of GLUT4 in heparin‐induced impairment of muscle glucose uptake. Consistently, while chronic heparin treatment led to hyperglycaemia, glucose intolerance and insulin resistance in control male BL6 mice, heparin failed to induce any of these changes in MCK‐GLUT4^−/−^ mice (Figure 3G‐K). Interestingly, we also found that chronic heparin treatment significantly increased body weight gain and food intake in both male BL6 and MCK‐GLUT4^−/−^ mice (Figure S3C,D). These results are consistent with our previous report on the orexigenic and obesogenic effects of heparin,^15^ and also suggest that heparin‐induced hyperglycaemia and hyperphagia may be mediated by distinct mechanisms. Taken together, our results suggest that heparin inhibits muscle GLUT4 translocation to decrease glucose uptake, and further induces hyperglycaemia and impaired glucose homeostasis.

### 3.4 Heparin reduces skeletal muscle glucose uptake by inhibiting the insulin signalling pathway

**FIGURE 4:**
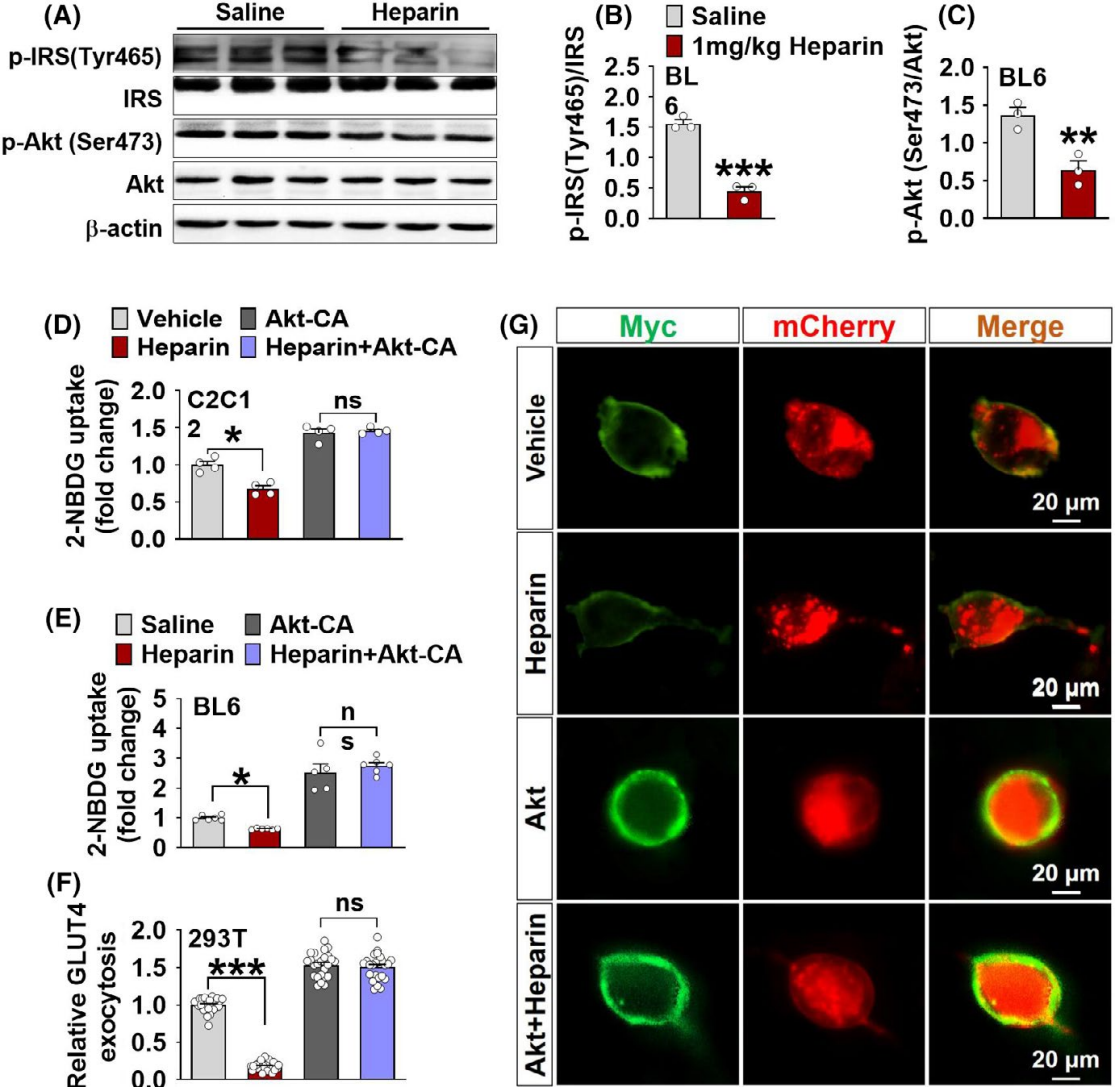
A‐C, Immunoblots (A) and quantification (B and C) of phosphorylation (at Tyr^465^) of insulin receptor substrate (IRS) and phosphorylation (at Ser^473^) of protein kinase B (Akt) in the GST of male BL6/J mice after i.p. injection of saline or 1 mg/kg heparin every other day for 16 days (*n* = 3). D, Uptake of 2‐NBDG in C2C12 cells transfected with constitutively active Akt or empty vehicle plasmid. Cells in each group were further divided into two groups to culture with vehicle or 100 μg/ml heparin for 12 h (*n* = 4), vehicle (cell culture medium). E, Uptake of 2‐NBDG in the GST of male BL6 mice injected with Akt CA or vehicle lentivirus into the GST. Mice in each group were further divided into two groups to receive i.p. injection of saline or 1 mg/kg heparin every other day for 16 days (*n* = 5 or 6). F‐G, Quantification (F) and representative images (G) of GLUT4 surface exposure in Myc‐GLUT4‐mCherry transfected 293T cells. The Myc‐GLUT4‐mCherry expressing 293T cells were transfected with constitutively active Akt plasmid and culture with vehicle or 100 μg/ml heparin for 12 h (*n* = 24 cells/each), vehicle (cell culture medium). Results are presented as mean ± SEM. *, *p* ≤ .05, **, *p* ≤ .01 or ***, *p* ≤ .001 in non‐paired Student's *t* test

Previously heparin has been shown to reduce the affinity of cells to insulin.^17^ Interestingly, skeletal muscle accounts for approximately 70% of insulin‐mediated glucose uptake.^24^ Based on these findings, we predict that heparin inhibits muscle glucose uptake through an insulin‐dependent pathway. In support of this view, we found that chronic heparin treatment significantly reduced the phosphorylation of two essential insulin cascade proteins (Figure 4A‐C), insulin receptor substrate (IRS) and Akt, in the GST of male BL6 mice, suggesting a heparin‐induced inhibition on the insulin signalling pathway. Consistent with these *in vivo* observations, heparin dose‐dependently inhibited the phosphorylation of IRS, Akt and phosphoinositide 3‐kinase (PI3K, an insulin receptor downstream enzyme that activates Akt) in *in vitro* C2C12 cells (Figure S4A‐D). These results indicate that heparin treatment in C2C12 cells exerts the same inhibitory effects on IRS/PI3K/Akt signalling pathways as in mice, which validated C2C12 cells as a model to study heparin’s effects on insulin signalling in skeletal muscle.

To directly test if PI3K/Akt mediates the inhibitory effects of heparin on muscle glucose uptake, we generated both *in vitro* and *in vivo* Akt overexpression models. In the *in vitro* model, we transfected C2C12 cells with a plasmid containing constitutively active Akt (Akt CA)^34^ to over‐express a constitutively phosphorylated (active) form of Akt mutant protein. In the *in vivo* model, we intramuscularly (i.m.) injected a lentivirus packed with Akt CA plasmid into the GST of male BL6 mice to selectively over‐express Akt CA protein in the skeletal muscle. We found that the heparin‐induced inhibition on glucose uptake was blocked in Akt CA transfected C2C12 cells (Figure 4D) as well as in the GST of male BL6 mice injected with Akt CA lentivirus (Figure 4E). Notably, heparin failed to induce any changes of glucose uptake in the EWAT and liver of both naïve and Akt CA‐infected male BL6 mice (Figure 4E). Thus, evidence from both strategies consistently indicates an essential role of PI3K/Akt in the inhibitory effects of heparin on muscle glucose uptake.

To further test if PI3K/Akt mediates the inhibitory effects of heparin on GLUT4 translocation, we adapted the strategies described above and applied them to Myc‐GLUT4‐mCherry plasmid transfected 293T cells. We found that Akt CA overexpression diminished the heparin‐induced decreases in the ratio of surface to total GLUT4 (Figure 4F,G). These results suggest a mediating role of PI3K/Akt in heparin‐induced inhibition of GLUT4 translocation. Therefore, our data support that heparin reduces muscle glucose uptake through inhibiting insulin‐dependent downstream cascades (IRS/PI3K/Akt/GLUT4).

### 3.5 Heparin impairs muscle glucose uptake by binding to insulin

**FIGURE 5:**
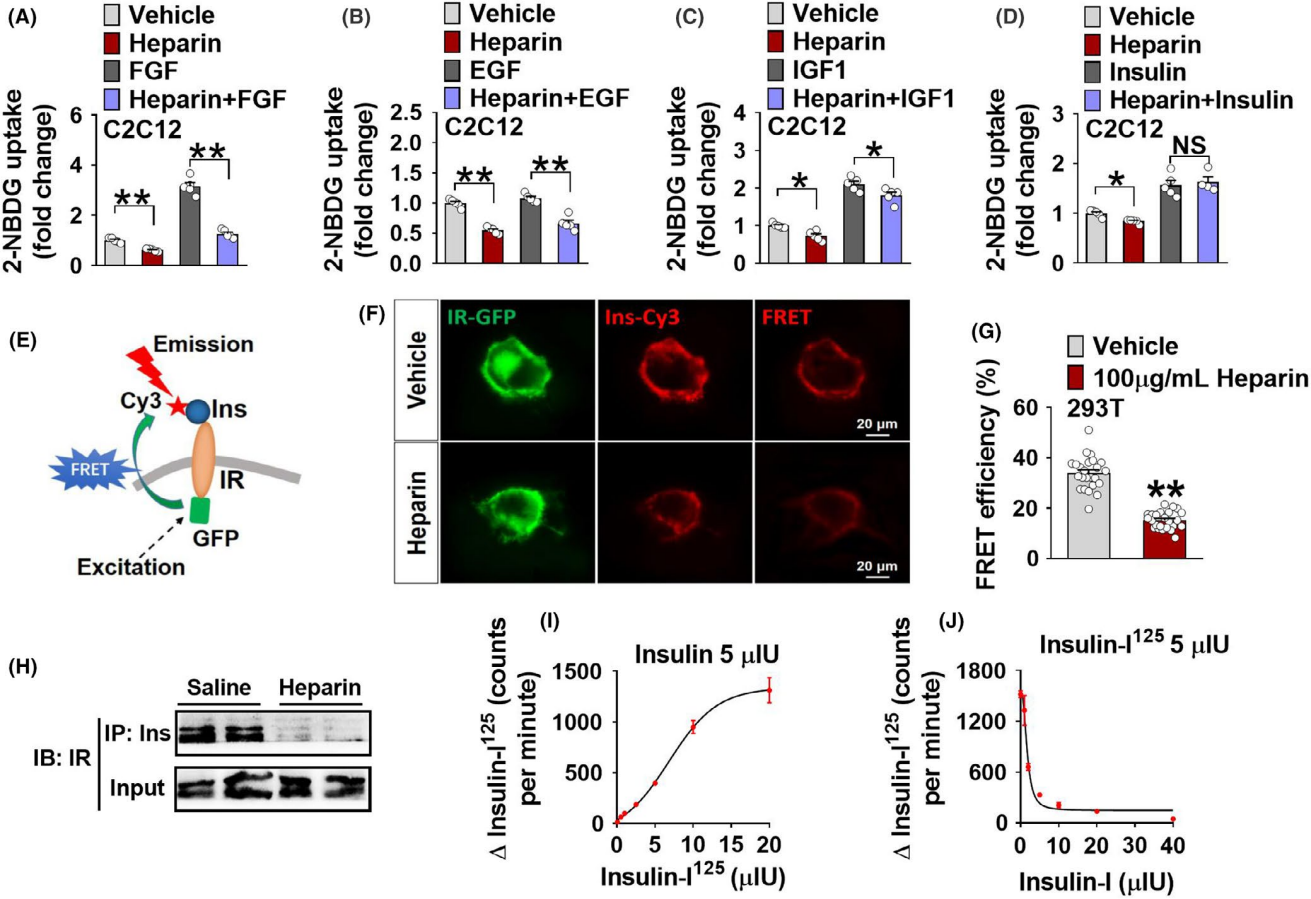
A, Uptake of 2‐NBDG in C2C12 cells cultured with vehicle, 100 μg/ml heparin, 10 ng/ml fibroblast growth factor (FGF) or heparin+FGF for 12 h (*n* = 5), vehicle (cell culture medium). B, Uptake of 2‐NBDG in C2C12 cells cultured with vehicle, 100 μg/ml heparin, 10 ng/ml epidermal growth factor (EGF) or heparin+EGF for 12 h (*n* = 5), vehicle (cell culture medium). C, Uptake of 2‐NBDG in C2C12 cells cultured with vehicle, 100 μg/ml heparin, 10 nM insulin‐like growth factor (IGF‐1) or heparin+IGF‐1 for 12 h (*n* = 5), vehicle (cell culture medium). D, Uptake of 2‐NBDG in C2C12 cells cultured with vehicle, 100 μg/ml heparin, 100 nM insulin or heparin+insulin for 12 h (*n* = 5), vehicle (cell culture medium). E, Schematic model of Insulin (Ins)‐insulin receptor (IR) intramolecular fluorescence resonance energy transfer (FRET) in IR‐GFP expressing 293T cells treated with insulin‐Cy3. FRET channel: Ex/Em:470/585 nm; GFP: donor; Cy3: acceptor. F‐G, Representative images (F) and FRET efficiency quantification (G) of the fluorescence intensities at the cell surface of IR‐GFP expressing 293T cells cultured with vehicle+Ins‐Cy3 or 100 μg/ml heparin+Ins‐Cy3 for 40 min. Sensitized emission was normalized to calculate FRET efficiency as described in Methods (*n* = 24), vehicle (cell culture medium). H, Immunoblot of IR in the immunoprecipitates of GST using anti‐Ins antibody. Male BL6/J mice were i.p. injected with saline or 1 mg/kg heparin every other day for 16 days. The GST was isolated and pulled down with anti‐Ins antibody. The associated IR was detected with anti‐IR antibody. IR in the immunoprecipitates was measured with immunoblotting. Input was measured by immunoblotting for IR in cell lysates. IP, Immunoprecipitation; IB, immunoblotting (*n* = 2). I, Scintillation counting in heparin beads incubated with increasing concentrations of insulin‐I125. Bead‐bound heparin agarose was incubated with 5 μIU insulin and radiolabelled insulin‐I125 at different concentration (0, 0.5, 1, 5, 10 and 20 μIU) at 4℃ for 19 h. After separation, the I125 radioactivity was detected in the bead‐bound heparin (*n* = 3). J, Scintillation counting in heparin beads incubated with increasing concentrations of insulin. Bead‐bound heparin agarose was incubated with 5 μIU radiolabelled insulin‐I125 and insulin at different concentration (0, 0.5, 1, 5, 10, 20 and 40 μIU) at 4℃ for 19 h. After separation, the I125 radioactivity was detected in the bead‐bound heparin (*n* = 3). Results are presented as mean ± SEM. *, *p* ≤ .05, **, *p* ≤ .01 in non‐paired Student's *t* test

Although PI3K/Akt/GLUT4 signalling is well‐known as an insulin‐dependent downstream pathway, it is also regulated by various other hormones, such as fibroblast growth factor (FGF),^35^ epidermal growth factor (EGF)^36^ and insulin‐like growth factor (IGF‐1).^37^ What is more, heparin did not decrease glucose uptake of C2C12 cells in serum‐free medium (Figure S5A), indicated that some of factors that regulate glucose uptake in serum were destroyed by heparin. To exclude the possibility that heparin acts through other hormone‐dependent signalling to reduce muscle glucose uptake, C2C12 cells were co‐cultured with heparin and insulin or other hormones. We found that the inhibitory effects of heparin on glucose uptake were only abolished by co‐treatment of insulin, but not FGF, EGF or IGF‐1 (Figure 5A‐D), suggesting a blockade of glucose uptake in C2C12 cells through reducing insulin‐dependent signalling.

We previously showed that heparin reduces food intake by inhibiting the binding of insulin to insulin receptor (IR) on agouti‐related peptide (AgRP) neurons in the arcuate nucleus of the hypothalamus (ARC).^15^ Based on these findings, we speculate a similar mechanism for the inhibitory effects of heparin on muscle glucose uptake. To test this possibility, 293T cells were transfected with a plasmid containing GFP‐IR^38^ and treated with insulin labelled with a Cy3 fluorescent probe, which is designed to be an acceptor for emission fluorescence released by GFP (Figure 5E). Intramolecular fluorescence resonance energy transfer (FRET) signals were used to represent insulin and IR binding, specifically quantified by fluorescence intensity at 585 nm when excited at 470 nm.^39^ We found that compared to control groups, that is 293T cells transfected with IR‐GFP without insulin‐Cy3 treatment or 293T cells treated insulin‐Cy3 without IR‐GFP transfection, FRET signals were significantly higher in IR‐GFP expressing 293T cells treated with insulin‐Cy3 (Figure S5B,C), which validated our model. We also found that FRET signals were significantly inhibited by heparin treatment (Figure 5F,G), suggesting reduced insulin‐IR binding. Consistently, we found that chronic heparin treatment reduced the expression of IR after immunoprecipitation of insulin in the GST of male BL6 mice (Figure 5H), suggesting a similar *in vivo* inhibitory effect induced by heparin. Our data from both *in vitro* and *in vivo* models consistently showed that heparin reduces binding of insulin to IR, which may mediate the inhibitory effects of heparin on muscle glucose uptake.

There are two possible mechanisms for heparin to interact with insulin and IR binding: it either directly binds with insulin or competes with insulin for IR binding. By using a heparin competitive binding assay, we showed that insulin dose‐dependently bound with heparin (Figure 5I,J). These observations are consistent with the previous report that heparin acts as a strong polyanion to bind with insulin and inhibit insulin aggregation.^40^ Our results suggest that heparin may directly bind with insulin to inhibit insulin/IR‐dependent downstream signalling pathways.

### 3.6 Insulin alleviates heparin‐induced hyperglycaemia

**FIGURE 6:**
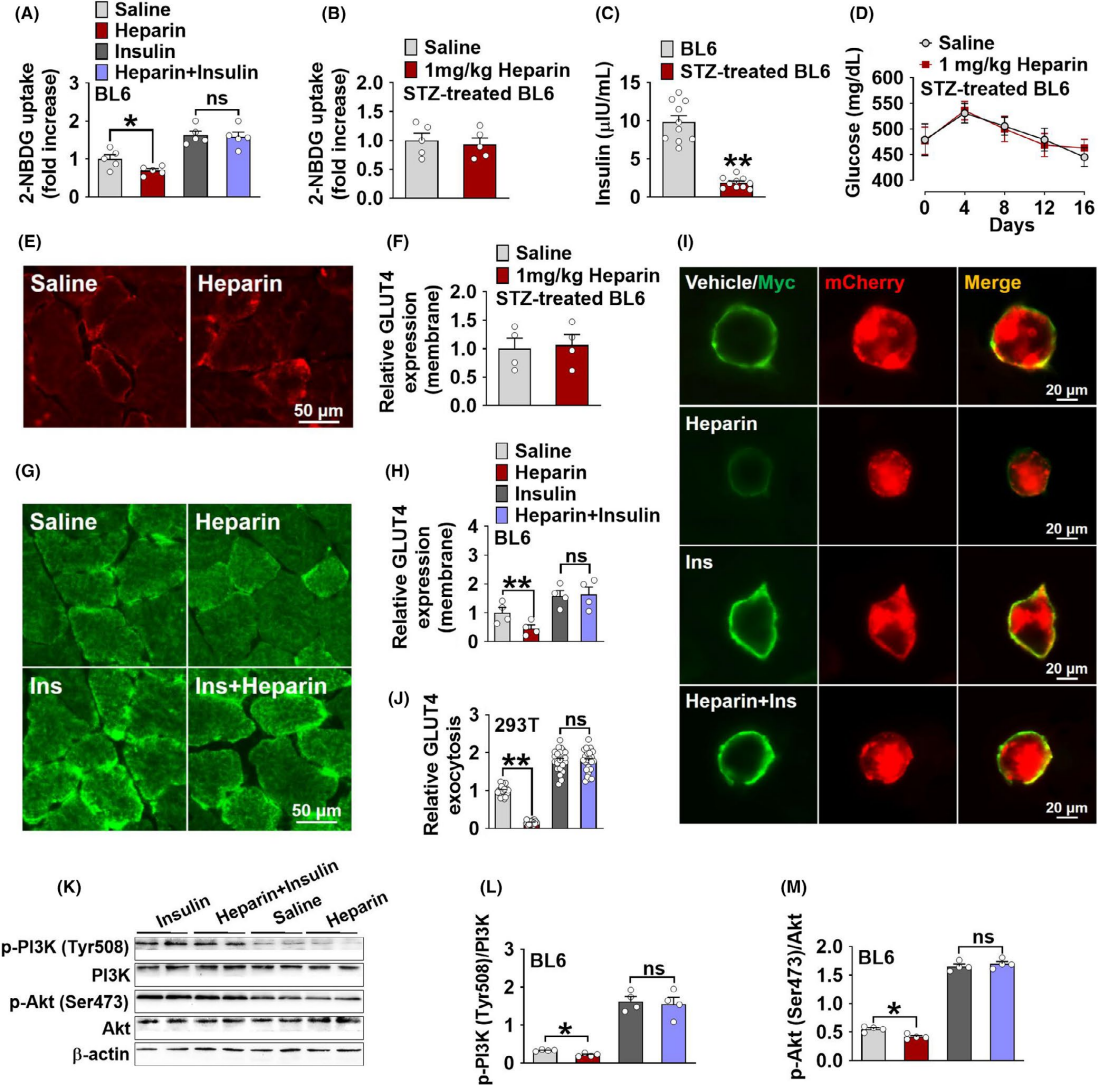
A, Uptake of 2‐NBDG in the GST of male BL6 mice. Male BL6 mice were i.p. injected with saline or 1 mg/kg heparin every other day for 16 days. At the end of heparin treatment, the GST was collected 40 min after i.p. injection of 2‐NBDG (2.5 mg/kg) or 2‐NBDG+insulin (1 U/kg) (*n* = 5). B, Uptake of 2‐NBDG in the GST of male BL6 mice treated with streptozotocin (STZ). At 8 weeks of age, male BL6 mice were i.p. injected with 60 mg/kg STZ once a day for 7 days. Two weeks after recovery, mice were i.p. injected with saline or 1 mg/kg heparin every other day for 16 days. At the end of heparin treatment, the GST was collected 40 min after i.p. injection of 2‐NBDG (2.5 mg/kg) (*n* = 5). C, Serum insulin levels in control or STZ‐treated male BL6 mice (*n* = 10). D. Blood glucose of STZ‐treated male BL6 mice i.p. injected with saline or 1 mg/kg heparin every other day for 16 days. (*n* = 5). E‐F, Representative images (E) and quantification (F) of GLUT4 immunofluorescent staining (red) in the GST of STZ‐treated male BL6 mice after 16 days of i.p. injection of saline or heparin (*n* = 4). G‐H, Representative images (G) and quantification (H) of GLUT4 immunofluorescent staining (green) in the GST of male BL6 mice. Male BL6 mice were i.p. injected with saline or 1 mg/kg heparin every other day for 16 days. At the end of heparin treatment, the GST of mice in each group was collected 40 min after i.p. injection of vehicle or 1 U/kg insulin (*n* = 4). I‐J, Representative images (I) and quantification (J) of GLUT4 surface exposure in Myc‐GLUT4‐mCherry expressing 293T cells cultured with vehicle, 100 μg/ml heparin, 100 nM insulin or heparin+insulin for 12 h (*n* = 24), vehicle (cell culture medium). K‐M, Immunoblots (K) and quantification (L and M) of phosphorylation of PI3K (at Tyr508) and phosphorylation of Akt (at Ser473) in the GST of male BL6 mice. Male BL6 mice were i.p. injected with saline or 1 mg/kg heparin every other day for 16 days. At the end of heparin treatment, the GST of mice in each mice was collected 40 min after i.p. injection of vehicle or 1 U/kg insulin (*n* = 4). Results are presented as mean ± SEM. *, *p* ≤ .05, **, *p* ≤ .01 in non‐paired Student's *t* test

To directly test if the suppression of insulin action is required for the heparin‐induced hyperglycaemia, we tested the inhibitory effects of heparin on muscle glucose uptake in mice with or without insulin treatment. In the male BL6 mice without insulin treatment, we found that heparin significantly decreased glucose uptake in the GST (Figure 6A). These results suggest that heparin impairs glucose homeostasis when a normal physiological dose of insulin is presented. Importantly, this inhibitory effect induced by heparin was blocked by pharmacological supplementation of 1 U/kg insulin (Figure 6A). This dose of insulin is a commonly used dose for insulin tolerance tests, which has been shown to effectively decrease blood glucose levels in normal lean mice.^41^ These observations clearly indicate an essential role of insulin suppression in heparin‐induced hyperglycaemia. This view is further supported by the evidence from a STZ‐induced T1DM mouse model. Specifically, we found that heparin failed to affect glucose uptake in the GST of STZ‐treated male mice (Figure 6B), which has very low circulating insulin level (Figure 6C). Consistently, 16 days of heparin treatment also failed to induce any changes of blood glucose (Figure 6D), suggesting an insulin‐dependent mechanism. Thus, it is clearly demonstrated that heparin induces hyperglycaemia by interrupting insulin’s functions.

Importantly, the changes of several important essential kinases/receptors in insulin‐dependent downstream cascades also support our hypothesis. For example, in STZ‐induced T1DM male mice, we found that chronic heparin treatment failed to affect GLUT4 translocation in the GST (Figure 6E,F). On the other hand, in the BL6 male mice, long‐term heparin treatment decreased GLUT4 translocation in the GST, and this heparin‐induced inhibition was diminished by acute insulin injection (Figure 6G,H). Consistent with these *in vivo* observations, we found a similar blockage effect of insulin on heparin‐induced decreases of GLUT4 translocation in the 293T cells (Figure 6I,J). Additionally, insulin also abolished the inhibitory effects of heparin on the phosphorylation of three essential insulin downstream protein kinases, IRS, PI3K and Akt, in both C2C12 cells (Figure S6A‐D) and the GST of BL6 male mice (Figure 6K‐M). Therefore, our data support a model that heparin binds with insulin to reduce the skeletal muscle glucose uptake and by doing so, induces hyperglycaemia and glucose homeostasis abnormalities.

## 4 DISCUSSION

The major finding of our study is that heparin, a widely used anticoagulant clinical drug, impairs skeletal muscle glucose uptake and disrupts glucose homeostasis in mice. Our study revealed that blood heparin levels are significantly increased in both pre‐diabetic (DIO) and diabetic (*db/db*) models in mice. Consistently, hyperglycaemia is associated with hyperheparinemia in humans. Long‐term systemic treatment of heparin induces hyperglycaemia, glucose intolerance and insulin resistance. We further provided both *in vitro* and *in vivo* evidence supporting that heparin induces hyperglycaemia by binding with insulin to inhibit the insulin‐dependent downstream cascade PI3K/Akt/GLUT4 signalling pathway, which impairs glucose uptake in skeletal muscle.

We recently found that systemic heparin treatment promotes weight gain and obesity by increasing food intake,^15^ suggesting an essential role of heparin in the regulation of energy homeostasis. It is well‐known that obesity is a major driving factor for diabetes. It is possible that heparin also plays an important role in the regulation of glucose homeostasis. Consistent with this view, we found that blood heparin concentrations increased in both DIO and *db/db* mice. DIO BL6 mice have been used as a model for pre‐diabetes, while *db/db* mice are the most commonly used T2DM model. Importantly, we also observed a positive association between levels of heparin and glucose in the blood of humans. This is consistent with previous findings that blood heparin levels are elevated in diabetic patients.^3^ Data from both animal and human studies consistently support a possible role of heparin in glucose homeostasis.

However, these association studies do not exclude the possibility that the increase in heparin levels is only a complication of diabetes development. To exclude this possibility and directly test the effects of heparin on glucose homeostasis, we systemically administered heparin, DSH and LMWH to normal chow‐fed BL6 mice. DSH is a heparin derivative with the 2‐O and 3‐O sulphate groups removed and lacks anticoagulant activity, whereas LMWH is a depolymerized form of heparin and produces more efficient anticoagulant activity.^42^ We found that chronic injection of heparin and DSH consistently induced hyperglycaemia, suggesting that heparin increases blood glucose independently of its anticoagulant effect. On the other hand, LMWH failed to induce any changes in blood glucose. Opposite to the hyperglycaemia phenotypes observed in heparin‐treated BL6 mice, we found that long‐term systemic treatment of heparinase, a heparin cleavaging enzyme, significantly decreased blood glucose in diabetic *db/db* mice, but not in normal chow‐fed BL6 mice. These findings are reminiscent of those observed in heparanase overexpression mice, which have decreased heparan sulphate,^22^ with decreased blood glucose levels in STZ‐induced diabetic condition, but not in normal physiological condition. This may be attributed to the fact that low levels of heparin are present in the blood under normal physiological condition, while hyperheparinemia is associated with diabetic conditions.

To further characterize the effects of heparin on glucose balance, we performed both glucose tolerance tests (GTT) and insulin tolerance tests (ITT). We found chronic heparin treatment‐induced glucose intolerance and insulin resistance. Importantly, these impairments were associated with increased blood insulin levels, suggesting that the heparin‐induced hyperglycaemia and glucose intolerance may be due to impaired insulin action. Interestingly, it has been shown that *in vitro*, heparin reduces insulin sensitivity of cultured human lymphocytes, but not adipocytes, erythrocytes or intact hepatoma cells.^17^ Our previous study also found that heparin decreases the insulin sensitivity of AgRP neurons in the ARC.^15^ These findings suggest that heparin may induce glucose homeostasis abnormalities by decreasing insulin action, possibly in a tissue‐specific way.

Glucose homeostasis is a dynamic process that is primarily dependent on the balance between glucose production by the liver and glucose consumption by insulin‐dependent tissues (such as skeletal muscle and adipose tissue) and insulin‐independent tissues (such as the liver).^43^ We found that chronic heparin treatment failed to affect the mRNA expressions of genes that are essential for gluconeogenesis and glycolysis in the liver of chow‐fed BL6 mice, suggesting unchanged glucose production and degradation. On the other hand, it was found that heparin dramatically decreased glucose uptake in the GST but not in the liver or EWAT, suggesting inhibitory effects of heparin on insulin‐dependent glucose consumption in skeletal muscle. It is notable that chronic heparin treatment also increased insulin concentration in the GST, suggesting higher insulin aggregation in skeletal muscle. A similar phenotype has been shown in T2DM patients with hyperinsulinemia, which is considered to be a defence mechanism to combat insulin insensitivity.^44^ Importantly, evidence from the C2C12 skeletal muscle cell line further revealed that heparin inhibits glucose uptake independently of its anticoagulant properties. Considering the fact that skeletal muscle is the predominant site of insulin‐mediated glucose uptake, and that skeletal muscle insulin resistance is the primary defect before overt hyperglycaemia develops,^45^ we speculate that heparin impairs glucose homeostasis by inducing skeletal muscle insulin resistance.

If heparin induces skeletal muscle insulin resistance, the insulin‐mediated downstream cascade would be inhibited by heparin administration. Consistently, we found that GLUT4 translocation (from the cytoplasm to cell membrane), which is one of the crucial insulin‐dependent downstream cascades, was inhibited by heparin in both C2C12 cells and GST. Based on these observations, we hypothesized that GLUT4 translocation is required for the inhibitory effects of heparin on skeletal muscle glucose uptake. To directly test this, we generated a mouse model with GLUT4 deleted selectively in the skeletal muscle. We found that these mutant mice showed inhibited skeletal muscle glucose uptake, which is consistent with previous studies,^46^ and indirectly validates our knockout model. Notably, although muscle‐specific GLUT4 KO mice have been reported to have normal body weight and muscle mass, these mice have reduced blood glucose, disrupted glucose homeostasis, decreased muscle contraction, and increased muscle fatigability.^47^ We can not exclude the possibility that these disruptions in muscle physiology impact the upstream mechanisms of glucose and insulin signalling. However, to the best of our knowledge, this is the best model at the current stage. Interestingly, we found that muscle‐specific knockout of GLUT4 diminished the hyperglycemic effects of heparin, without affecting the hyperphagic effects induced by heparin. These results suggest that two independent mechanisms are involved in the regulatory effects of heparin on appetite and skeletal muscle glucose uptake. To further support the essential role of insulin‐dependent cascades in heparin‐induced hyperglycaemia, we tested the role of GLUT4 upstream PI3K/Akt signalling. Specifically, we blocked heparin’s effects on PI3K/Akt signalling pathways by genetic activation of Akt. This strategy diminished the inhibitory effects of heparin on skeletal muscle glucose uptake. Our data support a model that heparin induces hyperglycaemia by inhibiting an insulin‐dependent downstream cascade and glucose uptake in the skeletal muscle.

Besides insulin, several other endocrine hormones (FGF, EGF and IGF‐1) have been shown to regulate PI3K/Akt signalling and enhance glucose uptake in skeletal muscle and fat tissue.^48^, ^49^, ^50^, ^51^, ^52^, ^53^, ^54^, ^55^ It is possible that heparin acts through FGF, EGF or IGF‐1‐dependent downstream signalling pathways to regulate glucose uptake in skeletal muscle. To exclude these possibilities, we tested the responses of C2C12 cells to the co‐treatment of heparin and four different hormones, that is insulin, FGF, EGF or IGF‐1. Supporting our hypothesized insulin‐dependent model, the inhibitory effects of heparin on glucose uptake were only blocked by insulin but not other hormones. Notably, insulin was also shown to diminish heparin‐induced inhibition on the PI3K/Akt/GLUT4 signalling pathway in C2C12 or 293T cells. Consistent with the *in vitro* evidence, systemic insulin administration abolished chronic heparin treatment‐induced inhibition on GLUT4 membrane expression and glucose uptake in skeletal muscle in male BL6 mice. These findings provide additional evidence to support that heparin induces hyperglycaemia through inhibiting insulin‐dependent glucose uptake in skeletal muscle.

Consistent with the previous reports,^15^, ^17^ we found that heparin reduced insulin binding to its receptor. These results suggest that heparin‐induced impairment of insulin and IR binding may contribute to the inhibitory effects of heparin on skeletal muscle glucose uptake. Notably, the molecular mechanism in which heparin interferes with insulin binding is still unknown. Previous studies have shown that both FGF and EGF have heparin‐binding sites,^56^, ^57^ which facilitate direct binding of heparin with FGF or EGF and subsequent activity regulation. However, insulin does not have any heparin‐binding sites (data not shown), suggesting an alternative mechanism. Importantly, heparin failed to induce any changes in GST GLUT4 membrane expression, GST glucose uptake and blood glucose in a STZ‐induced T1DM mouse model, which has partial destruction of the insulin‐producing β cells of the pancreas. On the other hand, in male BL6 mice, the detrimental effects of heparin on glucose homeostasis were abolished by insulin administration. These results clearly indicate that heparin impairs glucose homeostasis by inhibiting insulin action in skeletal muscle.

In conclusion, we found that independent of its anticoagulant functions, heparin plays a vital role in the regulation of insulin sensitivity and glucose homeostasis. We demonstrated that heparin interacts with insulin to inhibit insulin and IR binding, and subsequent insulin‐dependent downstream cascade in skeletal muscle, and by doing so, impairs skeletal muscle glucose uptake and glucose homeostasis. Thus, our findings identify heparin as a potential target for the treatment of diabetes. Our results also demonstrate that the clinical application of heparin in diabetic patients should be more cautious. Long‐term therapeutic beneficial effects of heparin on renal failure or cardiovascular diseases could be at the expense of increased glucose tolerance.

## 5 A STATEMENT IDENTIFYING THE GUARANTOR

Dr. Gang Shu, who is independent of any commercial funder or sponsor, is the ‘guarantor’ of the study. He had full access to all the data in the study and takes responsibility for the integrity of the data and the accuracy of the data analysis.

## 6 PRIOR PRESENTATION INFORMATION

The studies have not been presented in a scientific meeting or via webcast.

## CONFLICT OF INTEREST

The authors have declared that they have no conflict of interest.

## AUTHOR CONTRIBUTIONS

C. Z., Z. X., T. W. and Y. Y. carried out all data collection, analysis and manuscript writing. X. C., C. Y., P. X., L. W., X. Z., S. W., P. G., Q. X. and Y. Z. contributed to the conduct of the study. H. Y., N. P. and S. S. contributed to the manuscript writing and data interpretation. P. X., G. S. and Q. J. contributed to study design, data interpretation and manuscript writing.

## Supporting information

Supplementary Material

Fig S1

Fig S2

Fig S3

Fig S4

Fig S5

Fig S6

## Data Availability

The data that support the findings of this study are available from the corresponding author upon reasonable request.
