# Supplementary material for: Heparin impairs skeletal muscle glucose uptake by inhibiting insulin binding to insulin receptor
Source: Endocrinol Diabetes Metab. 2021 May 5;4(3):e00253. doi: 10.1002/edm2.253 (PMC8279624; doi:10.1002/edm2.253)
Supplement: Supplementary file 1 — Supplementary Material [file EDM2-4-e00253-s006.docx]

**Supplemental Material**

**Animals**

All experimental protocols and methods were conducted under the guidelines of The South China Agricultural University Center of Animal Science. All experiments were conducted in accordance with “The Instructive Notions with Respect to Caring for Laboratory Animals” issued by the Ministry of Science and Technology of the People’s Republic of China. C57BL/6 (BL6) mice were purchase from Animal Experiment Center of Guangdong Province (Guangzhou, Guangdong, China). Several transgenic mouse lines including MCK-Cre (006475, Jackson Laboratory), LSL-Cas9-EGFP (024857, Jackson Laboratory), and *db/db* mice (Shanghai Biomodel, China) were maintained on BL6 (MCK-Cre and LSL-Cas9-EGFP) or C57BLKS/J (BLKS, *db/db*) background.

**Cell culture**

Mouse myoblast C2C12 cells and HEK293T cells (both from ATCC, Manassas, VA, USA) were cultured in a high glucose DMEM (12800017, Thermo Fisher Scientific, Carlsbad, CA, USA) at 37 °C in a humidified atmosphere containing 5% CO2. The medium was supplemented with 10% FBS (16000044, Thermo Fisher Scientific), 100000 units/L penicillin sodium, and 100 mg/L streptomycin sulfate (11860038, Thermo Fisher Scientific). To initiate differentiation, when 80-90% confluency was reached, C2C12 cells were incubated in a high glucose DMEM containing 2% horse serum (16050122, Thermo Fisher Scientific).

**Serum heparin levels and correlation analysis**

At 10 weeks of age, male BL6 mice were divided into two groups receiving either a normal chow diet (5053, Guangdong Medical Science Experiment Center) or HFD (adjusted-calories diet that provided 60% of calories from fat, D12492, Guangdong Medical Science Experiment Center) for 4 weeks. At the end of experiment, mice were deeply anesthetized and euthanized. Serums were collected and used for spectrophotometric determination of serum heparin (Zhu et al., 2017). In another separate experiment, blood was collected from 14-week-old male *db*/+ and *db/db* mice fed with normal chow. Serums were separated for blood heparin measurement.

For the correlation analysis in humans, total of 122 volunteers (85 males, 37 females, aged from 30 to 60 years and body weight from 49.2 to 102.5 kg) were recruited and conducted in Huadong Sanatorium (Wuxi, China). All volunteers were required to complete a self-assessment form one week before samples collection, including age, gender, and symptoms of other diseases. Volunteers with other diseases that affect blood glucose level and diabetes treatment history were excluded. Each volunteer’s blood samples were collected and stored in EDTA tubes. Plasma samples were obtained by centrifugation (4°C, 4,000 × g for 20 min) and used for spectrophotometry detection of heparin and glucose. Whole procedure of this test was reviewed and approved by the Human Subjects Ethics Committee of Huadong Sanatorium, and got the written consent of each volunteer.

**Effect of heparin on glucose balance**

At 10 week old, male BL6 mice were randomly divided into two groups according to body weight. Either heparin (1mg/kg, S12004, Yuanyebio, Shanghai, China) or saline was i.p. injected every other day for 16 days. Glucose level were measured every four days using a glucometer (Yuwell, Guangzhou, China). GTT and ITT were performed at the end of the experiment. Mice were euthanized and blood samples were collected. Blood levels of insulin (80-INSMSU-E01, ALPCO, Boston, United State), corticosterone (H205, Njjcbio, Nanjin, China), and glucagon (H183, Njjcbio, Nanjin, China) were detected using ELISA kits per manufacturer's instructions. Gastrocnemius (GST) muscles were isolated for Western Blot detection of insulin pathway proteins and immunofluorescent staining of GLUT4. Additionally, the chronic effects of low molecular weight heparin (LMWH, Z140647, YuanYeBio, Shanghai, China) and desulfated heparin (DSH, DSH003/N, Yubiotech, Shanghai, China) on blood glucose were also tested following the same experimental procedures as described above. Male BL6 mice were i.p. injected with LMWH (1 mg/kg) or DSH (1 mg/kg) every other day for 16 days. At the end of experiment, blood glucose levels were measured.

**Effect of heparinase on blood glucose**

At 12 weeks of age, chow-fed male BL6 mice were i.p. injected with saline or 5 U/kg heparinase (H3917, Sigma, MO, USA) every other day for 16 days. Blood glucose levels were measured every 4 weeks. In a separate experiment, 12-weeks-old chow-fed male *db/db* mice were i.p. injected with saline or 5 U/kg heparinase every other day for 3 weeks. Blood glucose levels were recorded weekly.

**Heparin effects on glucose balance in STZ-treated mice**

At 8 weeks of age, male BL6/J mice were i.p. injected with 60 mg/kg STZ (S0130, Sigma, MO, USA) once a day for 7 days. Two weeks after injection, the blood glucose levels were measured. The mice with blood glucose levels higher than 450 mg/dl were considered diabetic and chosen for the following studies. Mice were randomly divided into two groups based on blood glucose levels. The selected mice received i.p. injections of 1 mg/kg heparin or saline every other day for 16 days. The blood glucose levels were recorded every four days. At the end of experiment, all mice were anesthetized and euthanized. The GST muscles were isolated for GLUT4 immunoflorescent staining. Serum was collected to detect blood insulin levels.

**Effect of heparin on *in vivo* 2-NBDG uptake**

Male C57BL6/J mice were i.p. injected with saline or 1 mg/kg heparin every other day for 16 days. Immediately after the last i.p. injection of heparin, mice were intravenous (i.v., tail vein) injected with 2.5 mg/kg 2-NBDG (N13195, Thermo Fisher Scientific). Forty minutes after injection, the mice were deeply anesthetized and euthanized, epididymal white adipose tissue (EWAT), GST, and liver were collected and lysed by RIPA buffer (P0013B, Beyotime Biotechnology, Shanghai, China). The fluorescence intensity of lysis was recorded at excitation and emission wavelengths of 485 nm and 535 nm respectively by fluorescence micro plate reader (Synergy 2, BioTek, Vermont, USA).

Additionally, another cohort of male BL6 mice were generated and i.p. injected with saline or 1 mg/kg heparin every other day for 16 days. On the last day of experiment, mice in each group were further divided to receive saline, 1 U/kg insulin (Eli Lilly, Indiana, USA), 1 mg/kg heparin, or 1 mg/kg heparin + 1 U/kg insulin. Immediately after the i.p. injection, all mice were i.v. injected with 2.5 mg/kg 2-NBDG. Forty minutes after the injection, the mice were deeply anesthetized and euthanized, GST was collected for 2-NBDG absorption analysis.

In another separate experiment, STZ-induced diabetic male BL6/J mice were generated as described before. These insulin-deficient mice were i.p. injected with saline or 1 mg/kg heparin every other day for 16 days. At the end of experiment, 2-NBDG absorption was detected in the GST.

To test if PI3K/Akt pathway is required for the inhibitory effects of heparin on glucose uptake, 7-week-old male BL6 mice were injected with vehicle or lentivirus packed with Akt constitutively active plasmid (12262 or 53583, Addgene, MA, USA; packed by Cyagen, Suzhou, China, 1 X 10^8^ TU/mL) into both side of GST. Four weeks after recovery, mice were i.p. injected with saline or 1 mg/kg heparin every other day for 16 days. At the end of experiment, 2-NBDG absorption was detected in the GST, EWAT, and liver.

**Effect of heparin on 2-NBDG uptake in C2C12 cells**

C2C12 cells were treated with vehicle or 100 μg/mL heparin in normal or serum-free medium for 12 hrs. After incubation, the supernatant medium was replaced by 100 μM 2-NBDG in PBS and incubated at 37°C for 40 min. Cells were then washed with ice-cold PBS three times and the fluorescence intensity was directly detected by fluorescence micro plate reader. Similarly, the effects of 100 μg/mL DS heparin and 100 μg/mL LMWH on 2-NBDG uptake in C2C12 were tested.

In another separate experiment, C2C12 cells were incubated with vehicle, 100 μg/mL heparin, 100 nM insulin (4772, biovision, California, USA), or 100 nM insulin + 100 μg/mL heparin for 12 hrs. At the end of incubation, 2-NBDG absorption was detected as described above. Similar, the effects of 10 ng/mL FGF (PMG0031, Thermo Fisher Scientific), 10 ng/mL EGF (E9644, Sigma, MO, USA), and 10 nM IFG-1 (P5502, Beyotime Biotechnology, Shanghai, China) on the heparin-induced inhibition on 2-NBDG uptake were tested in C2C12 cells.

To test if PI3K/Akt pathway is required for the inhibitory effects of heparin on glucose uptake, C2C12 cells were first transfected with empty vehicle plasmid or Akt constitutively active plasmid. Subsequently, the cells in each group were further divided into two groups receiving 12 hrs incubation of vehicle or 100 μg/mL heparin. At the end of incubation, 2-NBDG absorption was detected as described above.

**Effect of heparin on *in vivo* GLUT4 translocation**

Male BL6/J mice were i.p. injected with saline or 1 mg/kg heparin every other day for 16 days. At end of experiment, the mice were deeply anesthetized and euthanized. GST and liver were isolated and immediately frozen in liquid nitrogen. One aliquot of GST samples were lysed in RIPA buffer and used for Western Blot detection of GLUT4. The second aliquot of GST and liver samples were lysed in TRIzol Reagent (15596018, Thermo Fisher Scientific) and the total mRNA was extracted. The mRNA expression of glycolysis-related and glycolysis-related genes were quantified by RT-qPCR. The third aliquot of GST samples were frozen cut into 5 μm sections using a microtome (CM1510, Leica, Wetzlar, Germany). GST sections from 4 mice per group were fixed for 5 min in 75% acetone + 25% ethanol, following by 3 X 5 minutes washes in PBS. The sections were then incubated overnight with primary rabbit-anti-GLUT4 antibody (bs-0384R, Bioss, MA, USA) at room temperature in 5% normal goat serum, following by incubation in goat-anti-rabbit FITC conjugated secondary antibody (bs-0295G, Bioss) for 1 hr at room temperature. Sections were mounted on slides and cover slipped with DAPI mounting medium (0100-20, SouthernBiotech, AL, USA). Images were acquired by a Nikon fluorescence microscope (Ti-U, Nikon, Tokyo, Japan). The average fluorescence density of each cell membrane was analyzed with Image J software (NIH, Version 1.46). At least 3 sections from each mouse sample were used for analysis.

In another separate experiment, male BL6/J mice were i.p. injected with saline or 1 mg/kg heparin every other day for 16 days. On the last day of experiment, mice in each group were further divided to receive saline, 1 mg/kg heparin, 1 U/kg insulin, or 1 mg/kg heparin + 1 U/kg insulin. Forty minutes after injection, all mice were deeply anesthetized and euthanized. The GST samples were isolated and frozen cut into sections at 5 μm thickness. Sections from 4 mice per group were used for immunofluorescence detection of GLUT4 translocation as described above.

Similarly, STZ-induced diabetic male BL6/J mice were generated as described before. These insulin-deficient mice were i.p. injected with saline or 1 mg/kg heparin every other day for 16 days. At the end of experiment, GLUT4 translocation was quantified in the GST samples.

**Effect of heparin on GLUT4 translocation in 293T cells**

The 293T cells were transfected with Myc-GLUT4-mCherry (64049, Addgene, MA, USA) plasmid. Transfected 293T cells were treated with vehicle or 100 μg/mL heparin for 12 hrs. Cells were then collected and fixed by 4% paraformaldehyde for 10 minutes. Immunofluorescence staining was performed using rabbit-anti-Myc (bs-0384R, Bioss) at 4℃ overnight in 5% normal goat serum and FITC-conjugated goat-anti-rabbit secondary antibodies (bs-0295G, Bioss) at room temperature for 1 hr in non-permeabilized cells. The GLUT4 membrane translocation was quantified by the ratio between surface Myc fluorescence intensity and total GLUT4 mCherry fluorescence intensity using Image J software. At least 3 wells each group were used for analysis.

In another separate experiment, 293T cells were transfected with Myc-GLUT4-mCherry plasmid and cultured with vehicle, 100 μg/mL heparin, 100 nM insulin (4772, biovision, California, USA) or 100 μg/mL heparin + 100 nM insulin for 12 hrs. The GLUT4 membrane translocation was quantified as described above.

To test if the PI3K/Akt pathway is required for the inhibitory effects of heparin on GLUT4 translocation, 293T cells were transfected with Myc-GLUT4-mCherry plasmid and divided into 4 groups. Two groups were cultured with vehicle, 100 μg/mL heparin for 12 hrs. Other two groups of cells were transfected with constitutively active Akt plasmid and culture with vehicle or 100 μg/mL heparin for 12 hrs. The GLUT4 membrane translocation was quantified as described above.

**Heparin binding assay**

Bead-bound heparin agarose (ZY130413, Zeye, Shanghai, China) were incubated with radiolabeled insulin-I^125^ (5 μIU) (PID RM1, Jiuding, Tianjin, China) and different concentrations of insulin (0, 0.5, 1, 5, 10, 20, and 40 μIU) at 4 ℃ for 19 hours. After incubation with separating buffer (PID RM1, Jiuding, Tianjin, China), the mixture was centrifuged at 1,000 g for 10 minutes at 4 ℃. The supernatant was discard and the I^125^ radioactivity of the sediment was detected by gamma radioimmunoassay counter (GC-1500, Zonkia, Hefei, China). Background was subtracted (△CMP) to generate dissociation curves. One Site-Fit Ki equation was used for data analysis according to an established protocol (Chen et al., 2019).

In another separate experiments, bead-bound heparin agarose was incubated with 5 μIU insulin and radiolabeled insulin-I125 at different concentration (0, 0.5, 1, 5, 10, and 20 μIU) at 4℃ for 19 hours. After separation, the I125 radioactivity was detected in the bead-bound heparin as described above.

**Insulin and IR binding detected by FRET microscopy**

The 293T cells were transfected with an IR-GFP (22286, Addgene, MA, USA) (donor) plasmid and treated with vehicle + 100 nM insulin-Cy3 (acceptor) or 100 μg/mL heparin + 100 nM insulin-Cy3 for 40 minutes. The cells were then fixed by 4% paraformaldehyde and FRET was quantified as previously described (Verveer et al., 2006). Briefly, the fluorescence in the following channels was recorded using a Nikon fluorescence microscopy: donor to donor (Dd) channel (excitation (Ex)/emission (Em): 470/525 nm), acceptor to acceptor (Aa) channel (Ex/Em: 560/585 nm), and FRET (Da) channel (Ex/Em: 470 nm/585 nm). To calculate FRET efficiency, separate images of the donor only and the acceptor only samples (i.e. 293T cells transfected with IR-GFP without insulin-Cy3 treatment or 293T cells treated insulin-Cy3 without IR-GFP transfection) were acquired first. NIS-Elements software (Nikon) and sensitized emission method (Verveer et al., 2006) were used to calculate the corrected FRET efficiency.

**Skeletal muscle-specific knock out of GLUT4**

AAV virus vector carrying paired single guide RNAs (AAV1-sgRNAs-GLUT4) targeting GLUT4 (sgRNA binding sites with 205bp interval were located in intron 7 and exon 8 of the GLUT4 gene) was generated and packed by Cyagen (Suzhou, China). MCK-Cre mice were crossed with LSL-Cas9-EGFP mice to generate MCK-Cre/LSL-Cas9-EGFP (MCK-Cas9), a mouse model with Cas9 selectively over-expressed in MCK positive muscle cells. Three days after birth, male MCK-Cas9 mice were i.p. injected with AAV1-sgRNAs-GLUT4 (1×10^12^ GC /ml) to generate a muscle-specific GLUT4 deletion mouse model (MCK-GLUT4^-/-^). At 10 weeks of age, male MCK-GLUT4^-/-^ and control mice (LSL-Cas9-EGFP mice injected with AAV1-sgRNAs-GLUT4) were i.p. injected with saline or 1 mg/kg heparin every other day for 16 days. Glucose level, food intake, and body weight were measured every four days. At the end of experiment, OGTT and ITT were performed. Afterwards, half of the mice were use for 2-NBDG uptake assay to test glucose uptake in EWAT, GST, and liver as described before. Other half of the mice were euthanized. EWAT, GST, and liver were collected for Western Blot and genomic PCR tests of GLUT4.

**OGTT and ITT**

OGTT was performed after an overnight fasting. An oral gavage of 1 g/kg of glucose (G8270, Sigma, MO, USA) was given to the mice. Blood glucose levels were measured subsequently at 0, 20, 40, 60, 90, and 120 minutes after gavage. ITT was performed after 4 hours of fasting. The mice were i.p. injected with a single dose of insulin (1 U/kg). Blood glucose levels were measured at 0, 20, 40, 60, 90, 120 minutes after injection.

**Western Blot assay**

Total proteins were extracted from GST and C212 cells using RIPA lysis buffer (P0020, Solar bio, Beijing, China) containing 1 mM PMSF (P0100, Solar bio, Beijing, China). The concentration of protein was determined using a BCA protein assay kit (23225, Thermo Fisher Scientific). Western Blot was performed as we described before (Zhu et al., 2017). Briefly, equivalent amounts of protein (30 μg) were separated on 10% sodium dodecyl sulfate (SDS)–polyacrylamide gel electrophoresis gels. Goat-anti-p-IRS (sc-17194, Santa Cruz, TX, USA), rabbit-anti-IRS (3407, Cell signaling), rabbit-anti-p-Akt (4060, Cell signaling), rabbit-anti-Akt (9272S, Cell signaling), goat-anti-p-PI3K (sc-12929, Santa cruz), rabbit-anti-GLUT4 antibody (bs-0384R, Bioss, MA, USA), and rabbit-anti-PI3K (bs-0128R, Bioss) were incubated over night at 4 ℃, followed by incubation at room temperature for 1 hour with donkey-anti-goat or goat-anti-rabbit HRP conjugated secondary antibody (bs-0294D or bs-0295G, Bioss). Western Blots were visualized using SuperSignal West Pico Chemoluminescence substrate (34577, Thermo Fisher Scientific) and qualified by Image J software.

**Co-IP**

Total proteins from GST were extracted and quantified as described above. A total of 500 μg proteins in lysate were immunoprecipitated with antibodies specific to insulin (8138, Cell signaling, MA, USA) at 4 °C for overnight. Immune complexes were collected by incubation with a mixture of protein A- and G-Sepharose (P2006, Beyotime Biotechnology, Shanghai, China) at 4 °C for 6 hours. The immune complexes were washed three times before being eluted in 2× sodium dodecyl sulfate sample buffer. The protein expression of insulin receptor were then quantified by Western Blot (3020, Cell signaling).

**qPCR analyses**

Total mRNA from GST and liver were extracted using TRIzol Reagent (15596018, Thermo Fisher Scientific) according to the manufacturer’s instructions. After DNase I digestion (2270A, Takara Bio, Kusatsu, Shiga, Japan), the total mRNA was reverse-transcribed to cDNA using the M-MLV Reverse Transcriptase (M1705, Promega, Madison, WI, United State) and oligo (dT) 18 primer (3806, Takara Bio) according the instructions provided by the manufacturer. SYBR Green (DRR820A, Takara Bio) Real-Time PCR was performed according to published protocols (Bookout and Mangelsdorf, 2003). Results were normalized by the expression of house-keeping gene β-actin. The primer sequences are shown as follows: β-actin, S:5'-GGTCATCACTATTGGCAACGAG-3', A: 5'-GAGGTCTTTACGGATGTCAACG-3'; PFK, S: 5'-TGTGGTCCGAGTTGGTATCTT-3', A: 5'-GCACTTCCAATCACTGTGCC-3'; PK, S: 5'-GCCGCCTGGACATTGACTC-3', A: 5'-CCATGAGAGAAATTCAGCCGAG-3'; LDH, S: 5'-TGTCTCCAGCAAAGACTACTGT-3', A: 5'-GACTGTACTTGACAATGTTGGGA-3'; HK, S: 5'-CTGGGGAGACTAGCCCTGT-3', A: 5'-TGTCCCATAGTGTAGAGGTGATG-3'; GAPDH, S: 5'-CCATCACCATCTTCCAGGAG-3', A: 5'-ATGATGACCCTTTTGGCTCC-3'; PEPCK, S: 5'-CTGCATAACGGTCTGGACTTC-3', A: 5'-CAGCAACTGCCCGTACTCC-3'; PCX, S: 5'-CAGTGGCTGTCTACTCGGAG-3', A: 5'-CCGCATCTACACCATTTTCCT-3'; G6PC, S: 5'-CGACTCGCTATCTCCAAGTGA-3', A: 5'-GTTGAACCAGTCTCCGACCA-3'.

**Supplemental Data**

Figure S1


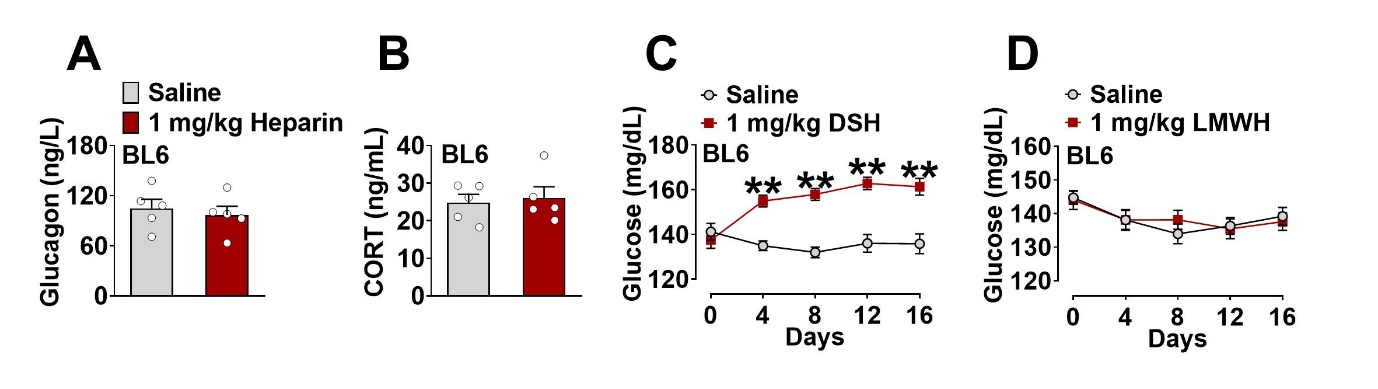
Supplementary figure 1. A-B. Serum glucagon (A) and corticosterone (B) levels in male BL6 mice after i.p. injection of saline or 1 mg/kg heparin every other day for 16 days (n=5).

C. Blood glucose of male BL6 mice after i.p. injection of saline or 1 mg/kg desulfated heparin (DSH) every other day for 16 days (n=5).

D. Blood glucose of male BL6 mice after i.p. injection of saline or low-molecular-weight heparin (LMWH) every other day for 16 days (n=6).

Results are presented as mean ± SEM. *, P≤0.05 in non-paired student's t test.

Figure S2


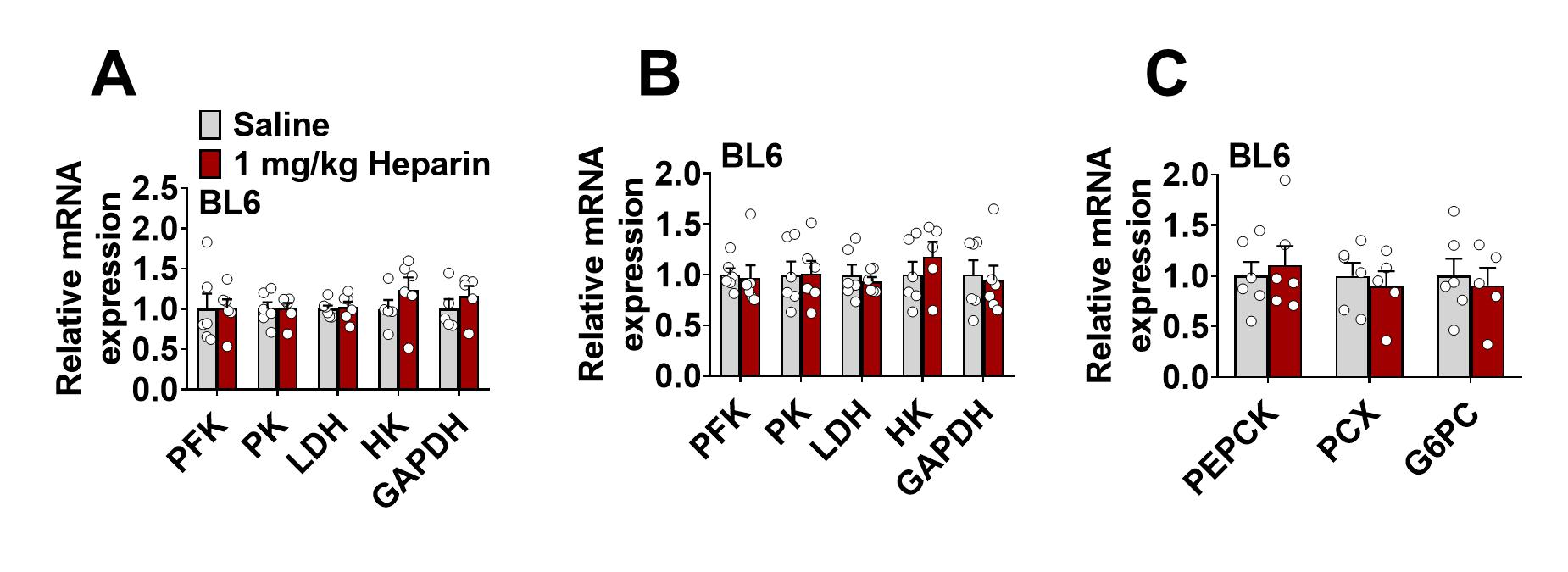


Supplementary figure 2. A. The mRNA expression of glycolysis-related genes in the GST of male BL6 mice after i.p. injection of saline or 1 mg/kg heparin every other day for 16 days (n=5 or 6). PFK, phosphofructokinase; PK, pyruvate kinase; LDH, lactate dehydrogenase; HK, hexokinase; GAPDH, glyceraldehyde-3-phosphate dehydrogenase.

B. The mRNA expression of glycolysis-related genes in the liver of male BL6 mice after i.p. injection of saline or 1 mg/kg heparin every other day for 16 days (n=5 or 6).

C. The mRNA expression of gluconeogenesis-related genes in the liver of male BL6 mice after i.p. injection of saline or 1 mg/kg heparin every other day for 16 days (n=5 or 6). PEPCK, phosphoenolpyruvate carboxykinase; PCX, pyruvate carboxylase; G6PC, glucose 6-phosphatase.

Results are presented as mean ± SEM and analyzed by non-paired student's t test.

Figure S3


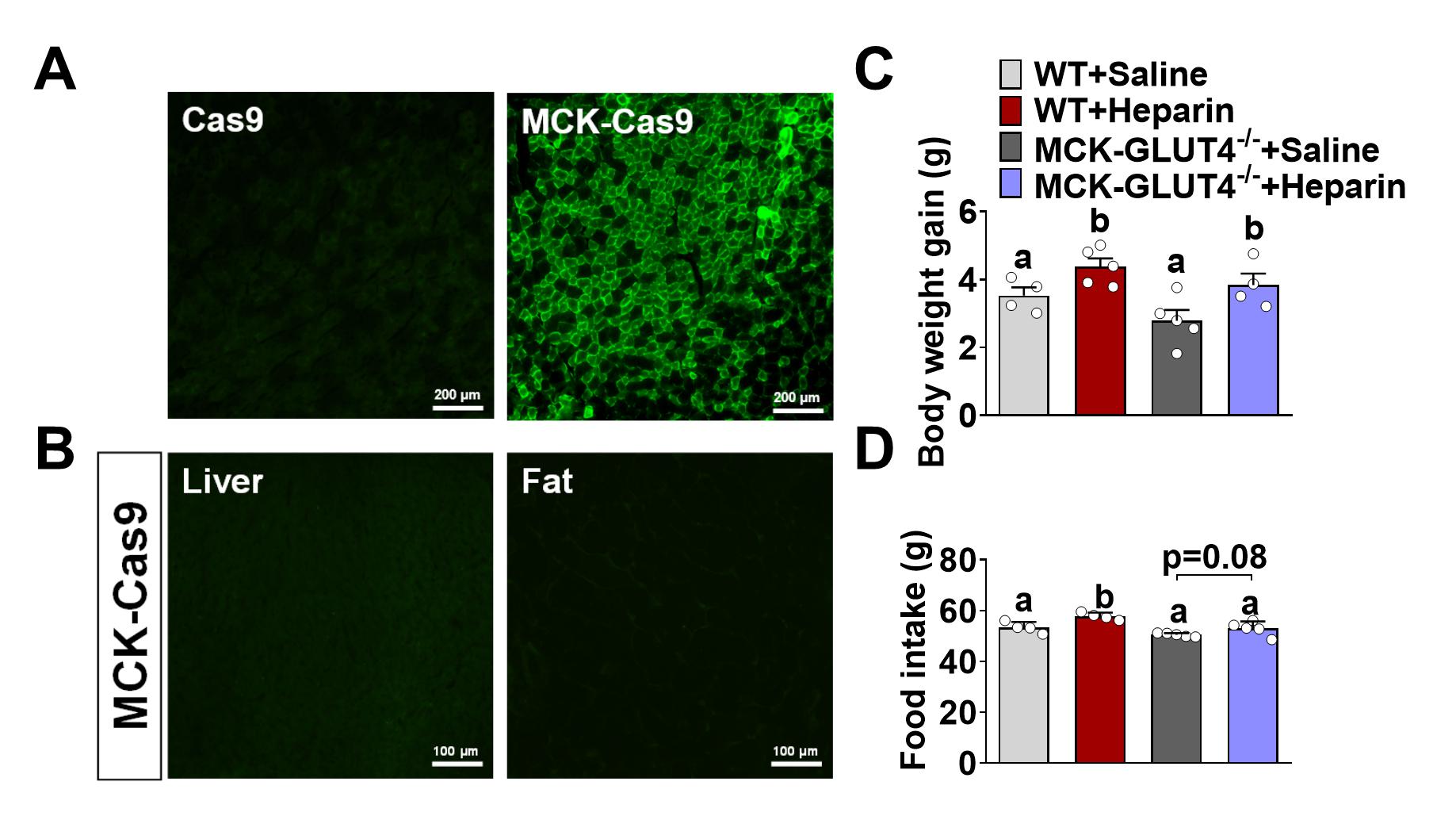


Supplementary figure 3. A: Expression of GFP in muscle of Cas9 and MCK-Cas9 mice.

B: Expression of GFP in liver and fat of MCK-Cas9 mice.

C-D. Body weight gain (C) and food intake (D) of male BL6 control or MCK-GLUT4^-/-^ mice after i.p. injection of saline or 1 mg/kg heparin every other day for 16 days (n=4 or 5).

Results are presented as mean ± SEM, different letters between bars indicate p≤0.05 by one-way ANOVA followed by post hoc Tukey’s tests.

Figure S4


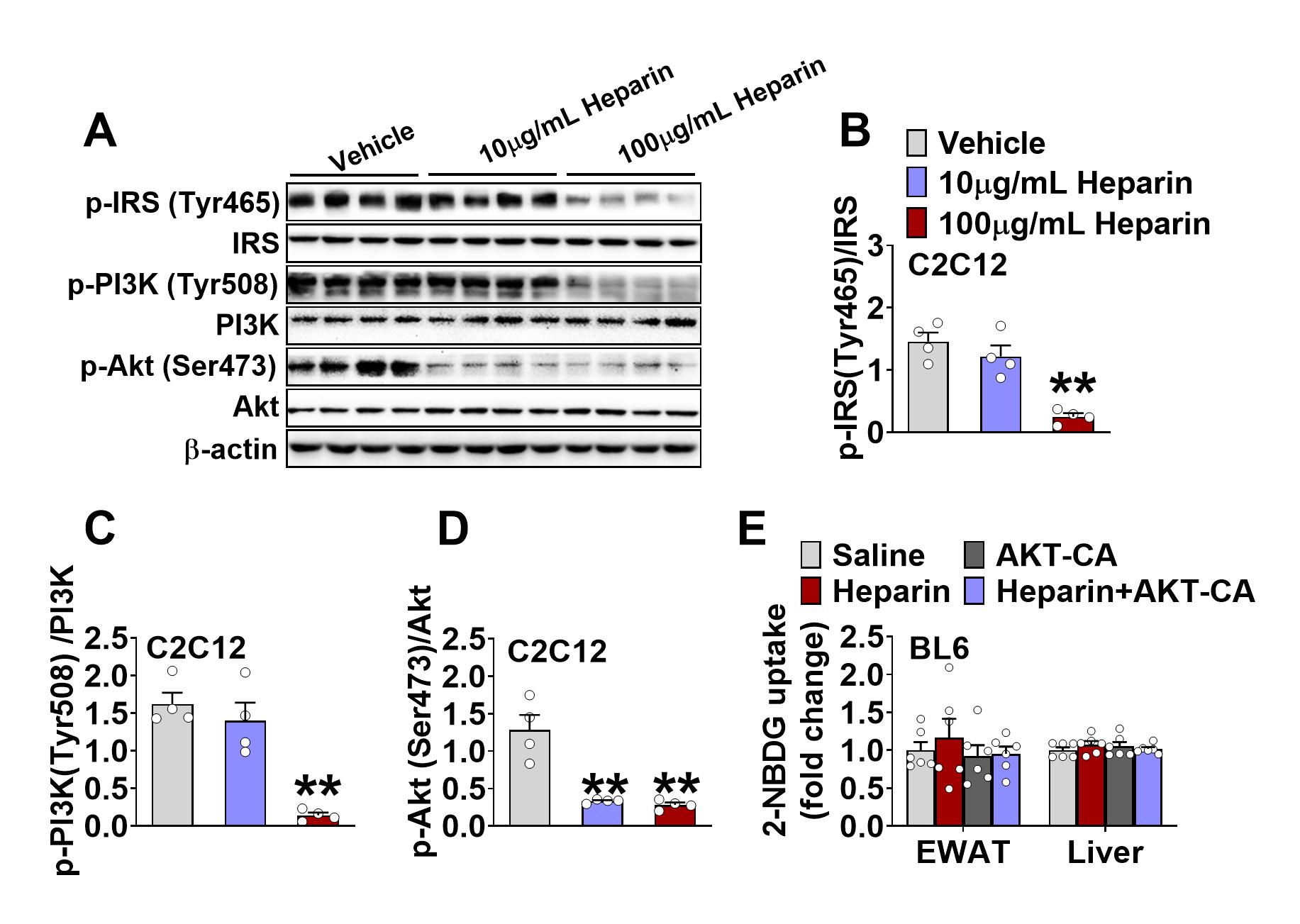


Supplementary figure 4. A-D. Immunoblots (A) and quantification (B-D) of phosphorylation of IRS (at Thy465), PI3K (at Tyr508), and Akt (at Ser473) in C2C12 cells cultured with vehicle, 10 μg/mL and 100 μg/mL heparin for 12 hrs (n=4), vehicle (cell culture medium).

E. Uptake of 2-NBDG in the EWAT and liver of male BL6 mice injected with Akt-CA or vehicle lentivirus into the GST. Mice in each group were further divided into two groups to receive i.p. injection of saline or 1 mg/kg heparin every other day for 16 days (n=5 or 6).

(B-D) Results are presented as mean ± SEM. **, P≤0.01 in non-paired student's t test. (E) Results are presented as mean ± SEM, different letters between bars indicate p≤0.05 by one-way ANOVA followed by post hoc Tukey’s tests.

Figure S5


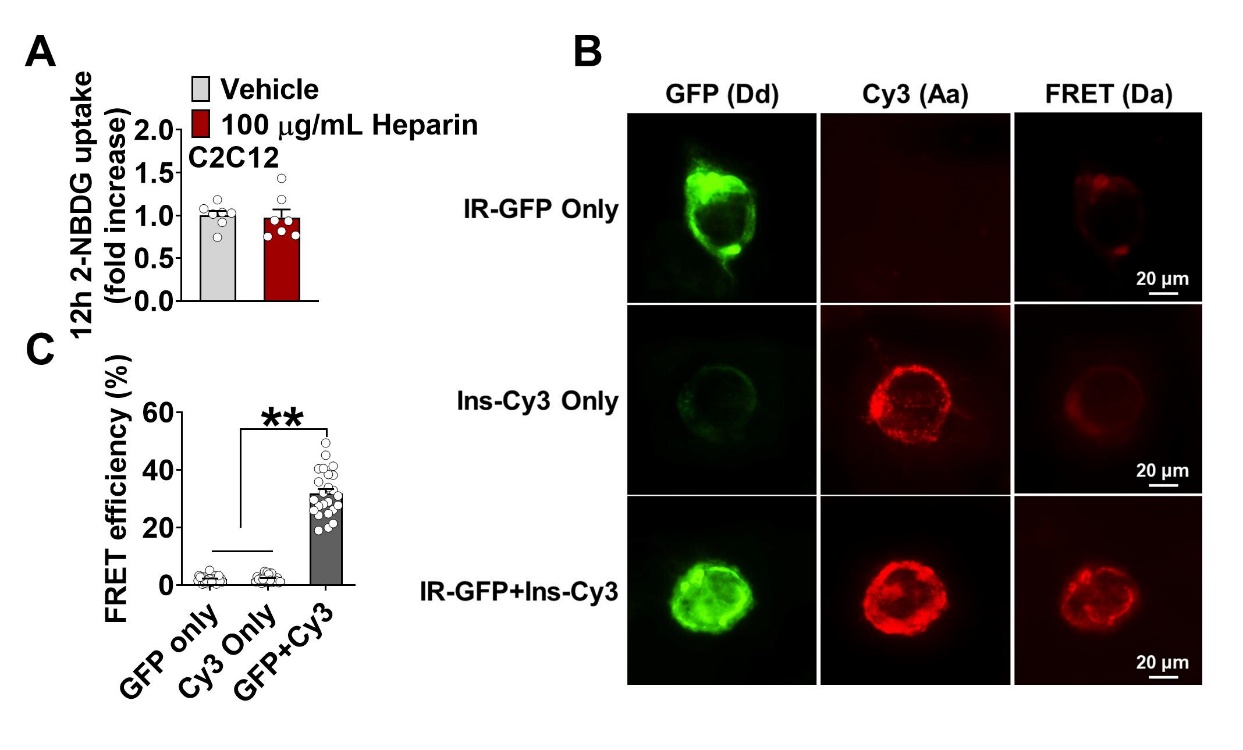


Supplementary figure 5. A. Uptake of 2-NBDG in C2C12 cells cultured with vehicle or 100 μg/mL heparin for 12 hrs in serum-free medium (n=7). B-C. Representative images (B) and FRET efficiency quantification (C) of the ﬂuorescence intensities at the cell surface of 293T cells transfected with IR-GFP plasmid only, cultured with insulin-Cy3 only or transfected with IR-GFP plasmid and cultured with insulin-Cy3 for 40 minutes. Sensitized emission was normalized to calculate FRET efficiency as described in Methods (n=24). Dd: Donor to donor channel; Aa: Acceptor to acceptor channel; Da: Donor to acceptor channel.

Results are presented as mean ± SEM. **, p≤0.01 in non-paired student's t test.

Figure S6


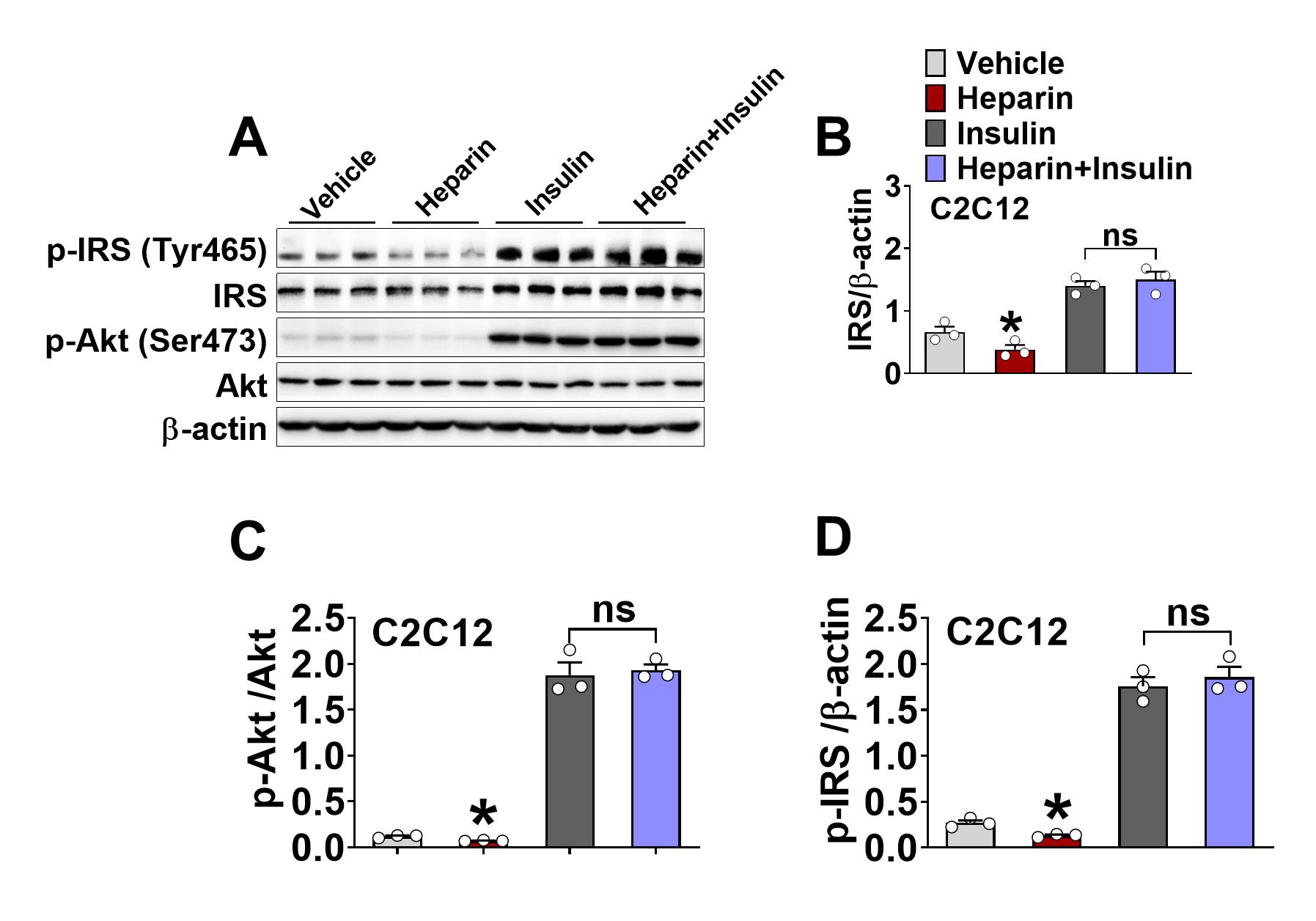


Supplementary figure 6. A-D. Immunoblots (A) and quantification (B-D) of phosphorylation of IRS (at Tyr465) and phosphorylation of Akt (at Ser473) in C2C12 cells cultured with vehicle, 100 μg/mL heparin, 100 nM insulin, or heparin+insulin for 12 hrs (n=3), vehicle (cell culture medium).

Results are presented as mean ± SEM. *, p≤0.05 in non-paired student's t test.
