# Supplementary figures and images for: Heparin impairs skeletal muscle glucose uptake by inhibiting insulin binding to insulin receptor
Source: Endocrinol Diabetes Metab. 2021 May 5;4(3):e00253. doi: 10.1002/edm2.253 (PMC8279624; doi:10.1002/edm2.253)

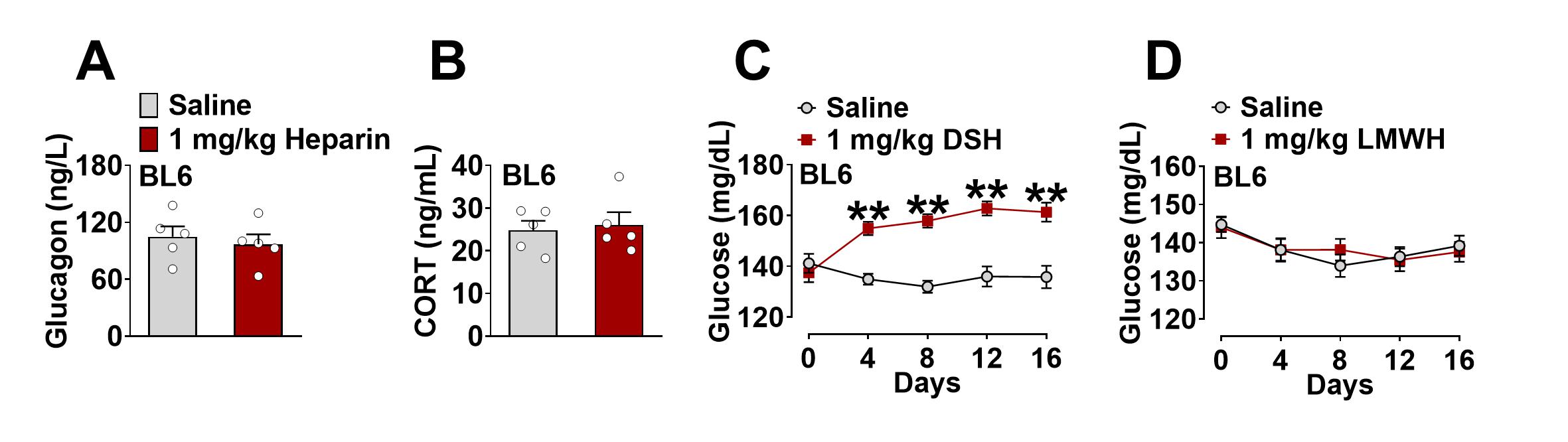

Supplement: Supplementary file 2 — Fig S1 [file EDM2-4-e00253-s004.jpg]

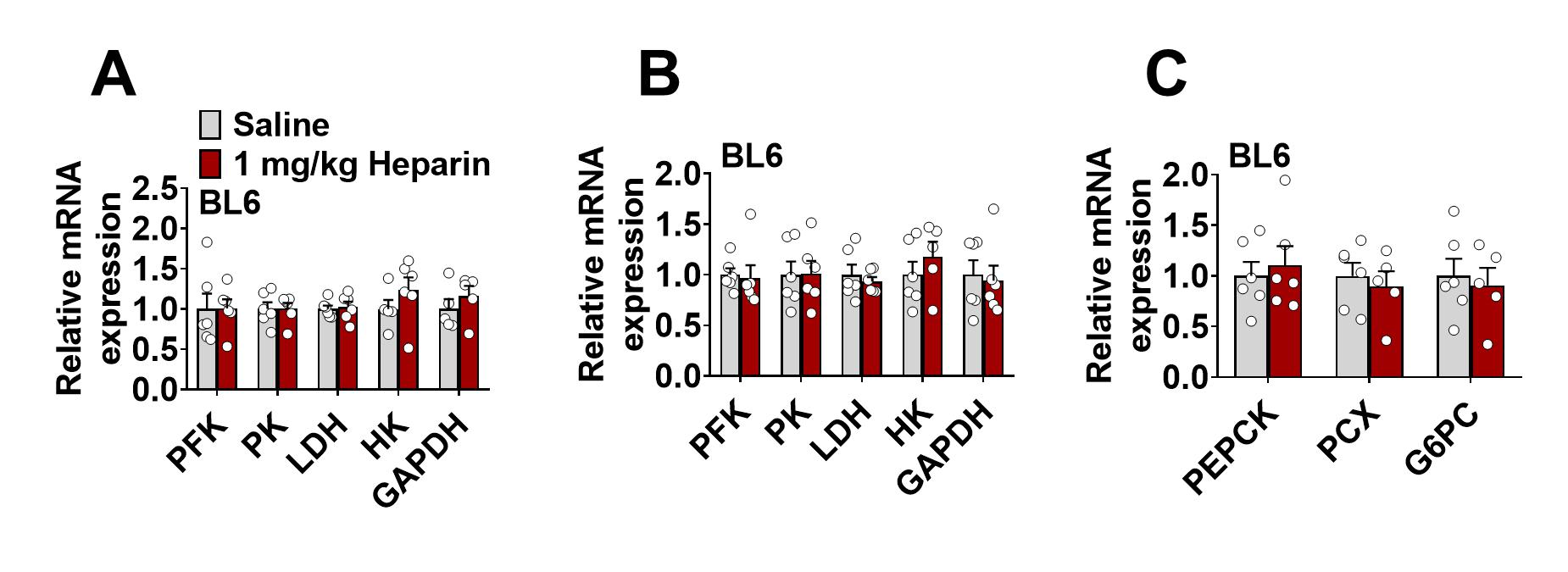

Supplement: Supplementary file 3 — Fig S2 [file EDM2-4-e00253-s001.jpg]

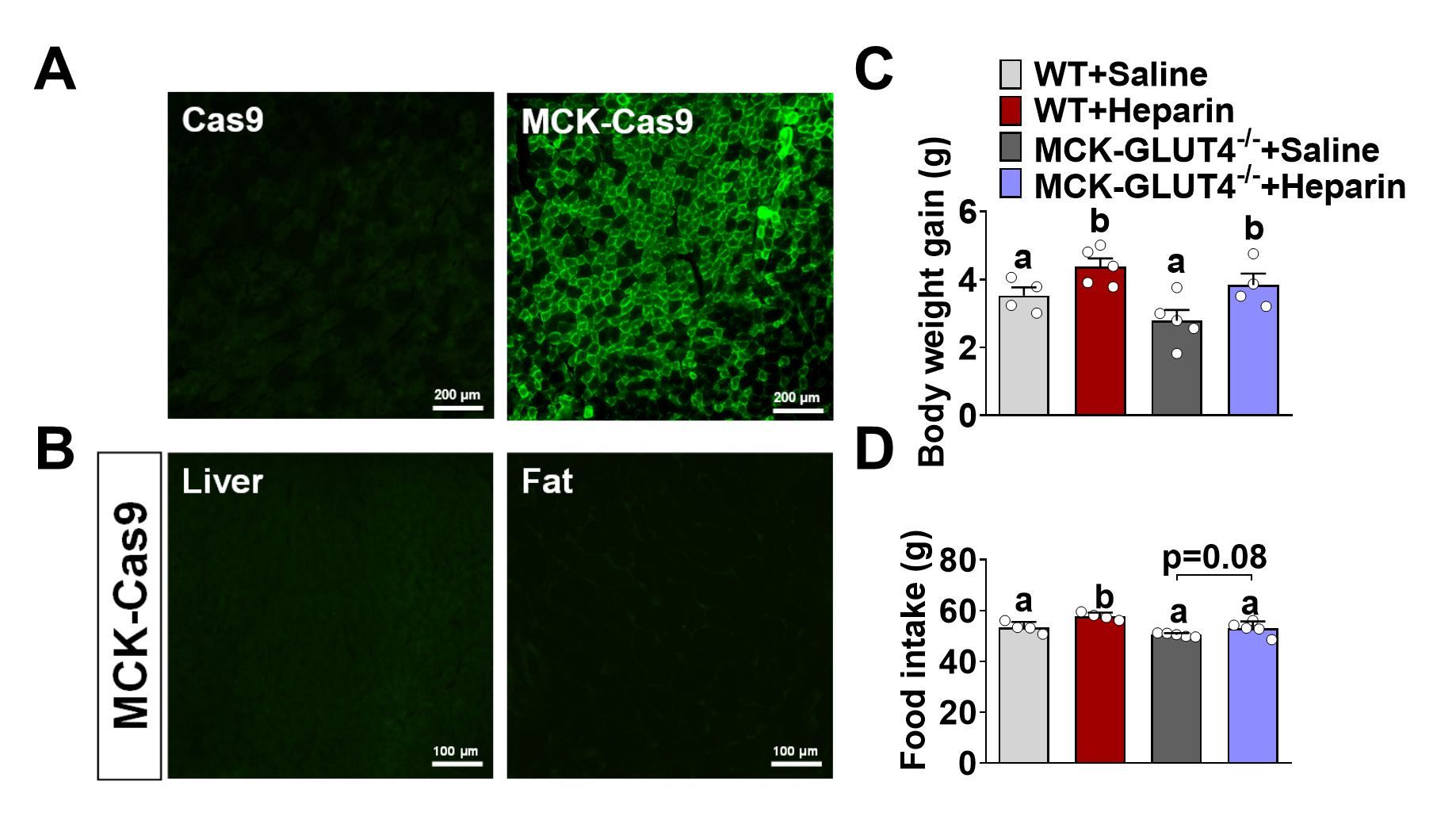

Supplement: Supplementary file 4 — Fig S3 [file EDM2-4-e00253-s007.jpg]

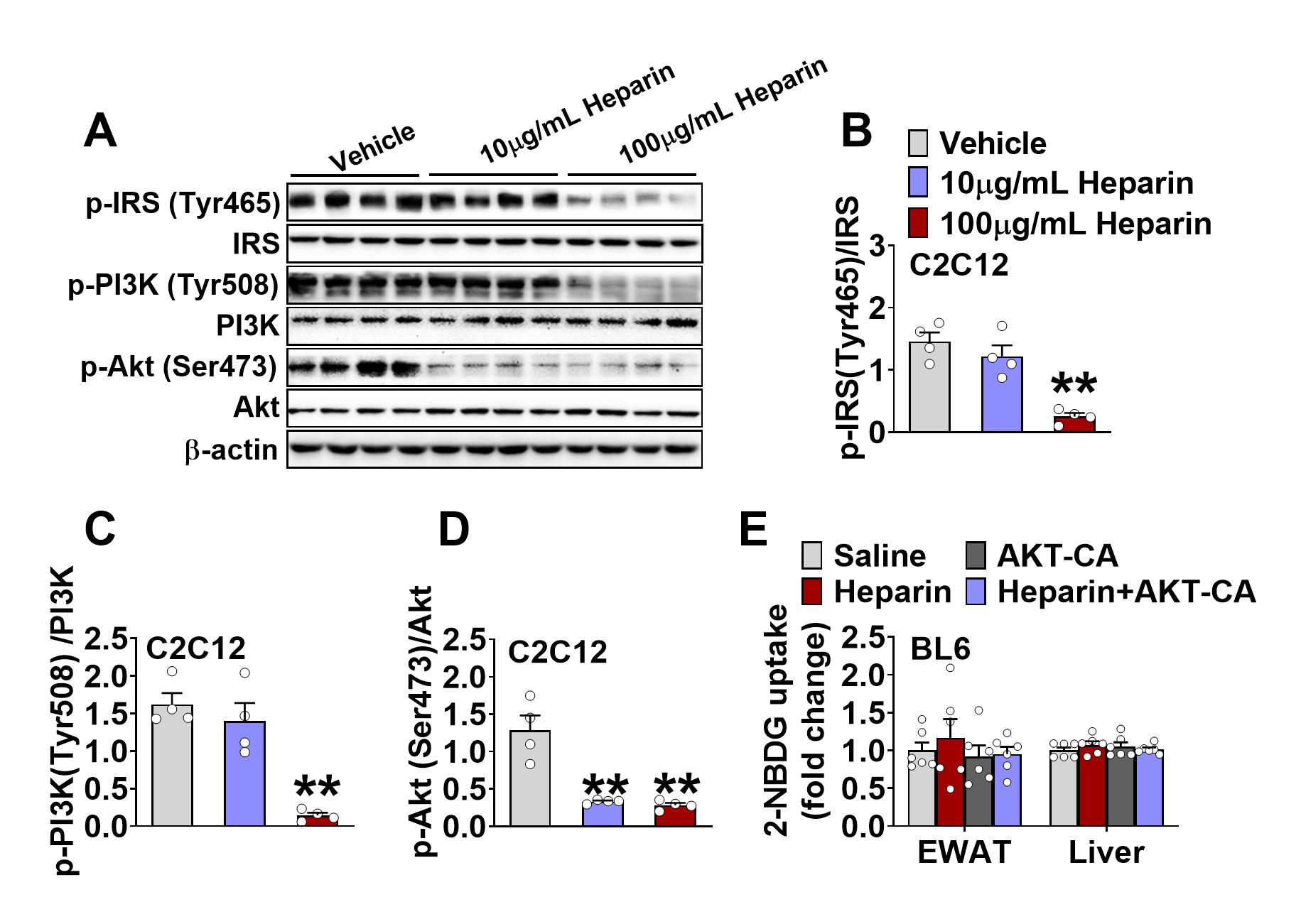

Supplement: Supplementary file 5 — Fig S4 [file EDM2-4-e00253-s003.jpg]

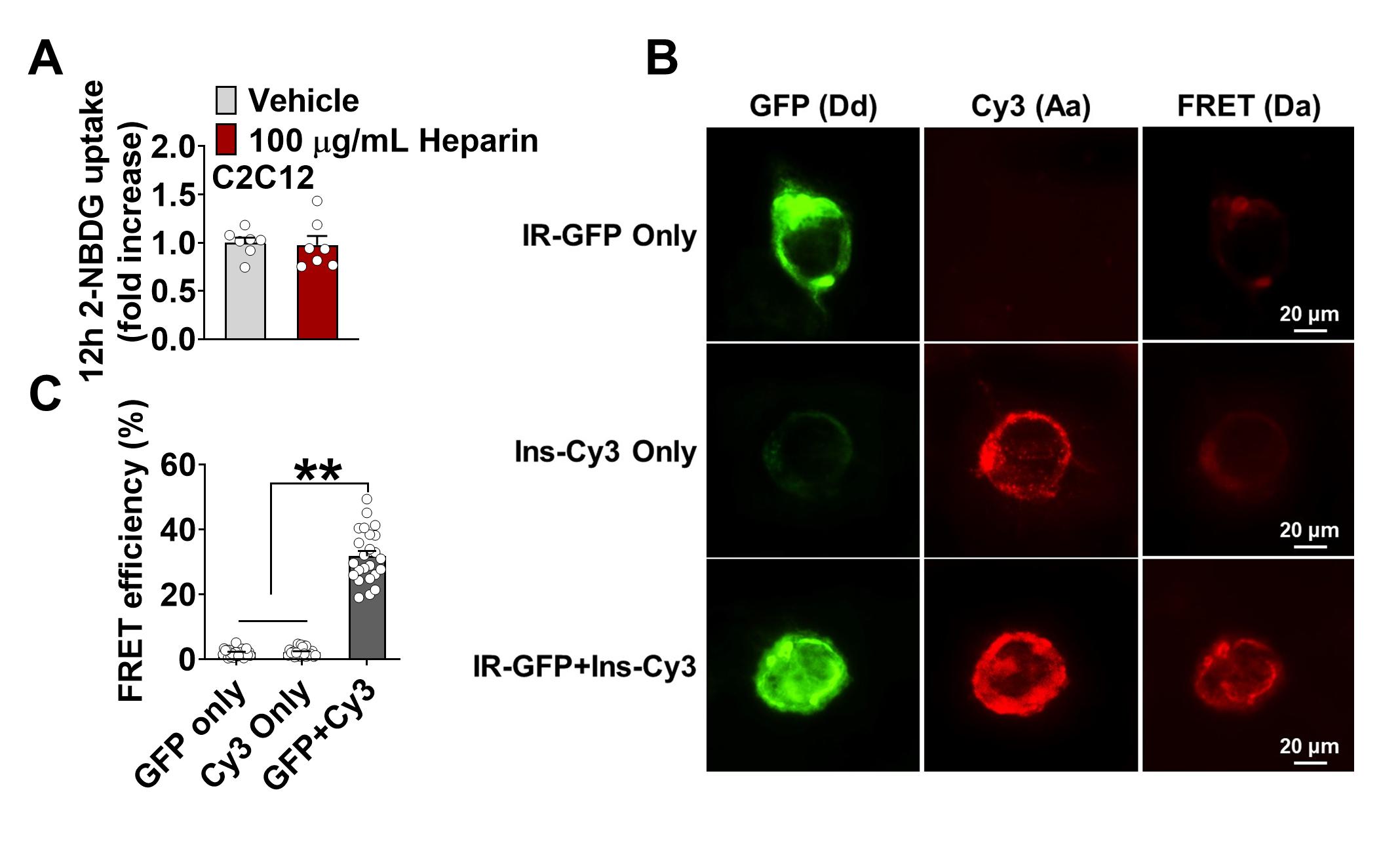

Supplement: Supplementary file 6 — Fig S5 [file EDM2-4-e00253-s005.jpg]

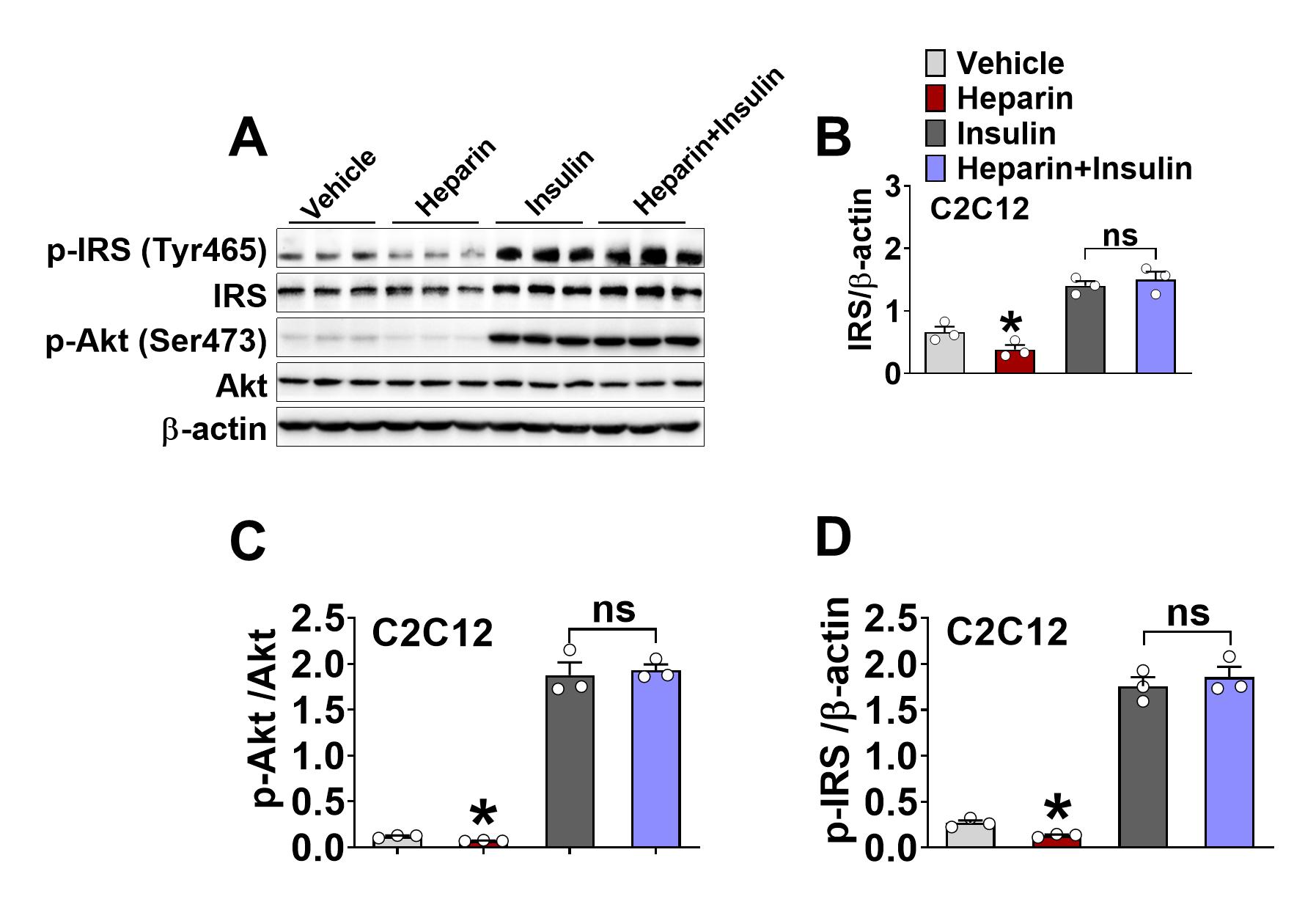

Supplement: Supplementary file 7 — Fig S6 [file EDM2-4-e00253-s002.jpg]
